# Nationwide incidence of sarcomas and connective tissue tumors of intermediate malignancy over four years using an expert pathology review network

**DOI:** 10.1371/journal.pone.0246958

**Published:** 2021-02-25

**Authors:** Gonzague de Pinieux, Marie Karanian, Francois Le Loarer, Sophie Le Guellec, Sylvie Chabaud, Philippe Terrier, Corinne Bouvier, Maxime Batistella, Agnès Neuville, Yves-Marie Robin, Jean-Francois Emile, Anne Moreau, Frederique Larousserie, Agnes Leroux, Nathalie Stock, Marick Lae, Francoise Collin, Nicolas Weinbreck, Sebastien Aubert, Florence Mishellany, Celine Charon-Barra, Sabrina Croce, Laurent Doucet, Isabelle Quintin-Rouet, Marie-Christine Chateau, Celine Bazille, Isabelle Valo, Bruno Chetaille, Nicolas Ortonne, Anne Brouchet, Philippe Rochaix, Anne Demuret, Jean-Pierre Ghnassia, Lenaig Mescam, Nicolas Macagno, Isabelle Birtwisle-Peyrottes, Christophe Delfour, Emilie Angot, Isabelle Pommepuy, Dominique Ranchere, Claire Chemin-Airiau, Myriam Jean-Denis, Yohan Fayet, Jean-Baptiste Courrèges, Nouria Mesli, Juliane Berchoud, Maud Toulmonde, Antoine Italiano, Axel Le Cesne, Nicolas Penel, Francoise Ducimetiere, Francois Gouin, Jean-Michel Coindre, Jean-Yves Blay, on behalf of the NetSarc/RePPS/ResSos and French Sarcoma Group- Groupe d’Etude des Tumeurs Osseuses (GSF-GETO) networks

**Affiliations:** 1 Department of pathology, CHU de Tours, Tours, France; 2 Department of Biopathology, Centre Léon Bérard, Lyon, France; 3 Department of Biopathology, Institut Bergonié, Bordeaux, France; 4 Department of Biopathology, Institut Claudius Regaud et Institut Universitaire du Cancer de Toulouse—Oncopôle, Toulouse, France; 5 Department of Biopathology, Gustave Roussy, Villejuif, France; 6 Department of pathology, La Timone University Hospital, Marseille, France; 7 Pathology Department, Saint-Louis Hospital, AP-HP, Université de Paris, Paris, France; 8 Pôle de Biologie-Pathologie-Génétique Centre Oscar Lambret, & Institut de Pathologie entre Oscar Lambret & CHU Lille, Lille, France; 9 Department of Biopathology, Hopital Ambroise Paré, Boulogne, France; 10 Department of Pathology, Department of Orthopedy CHU Nantes, Nantes, France; 11 Department of Biopathology, Hôpital Cochin-Saint-Vincent de Paul, Paris, France; 12 Department of Biopathology, Institut de Cancérologie de Lorraine—Alexis Vautrin, Vandoeuvre-lès-Nancy, France; 13 Department of Biopathology, Eugene Marquis Comprehensive Cancer Center & CHU Rennes, Rennes, France; 14 Department of Biopathology, Institut Curie, Paris, France; 15 Department of Biopathology, Centre Georges François Leclerc, Dijon, France; 16 Medipath, Frejus, France; 17 Department of Biopathology, Centre Jean Perrin, Clermont-Ferrand, France; 18 Department of pathology, CHRU Brest, Brest, France; 19 Department of Biopathology, Institut de Cancérologie de Montpellier & CHU Montpellier, Montpellier, France; 20 Department of Biopathology, Centre Francois Baclesse, Caen, France; 21 Department of Pathology, Institut de Cancerologie de l’Ouest, Angers, France; 22 Department of Biopathology, Hopital Henri Mondor, Creteil, France; 23 Department of Biopathology, Centre Paul Strauss, Strasbourg, France; 24 Department of Biopathology, Institut Paoli Calmettes, Marseille, France; 25 Department of Biopathology, Centre Antoine-Lacassagne, Nice, France; 26 Department of Biopathology, CHU Limoges, Limoges, France; 27 Department of Medicine of Centre Leon Berard, University Claude Bernard Lyon I, Lyon, France; 28 Headquarters, Unicancer, Paris, France; Fondazione IRCCS Istituto Nazionale dei Tumori, ITALY

## Abstract

**Background:**

Since 2010, nationwide networks of reference centers for sarcomas (RREPS/NETSARC/RESOS) collected and prospectively reviewed all cases of sarcomas and connective tumors of intermediate malignancy (TIM) in France.

**Methods:**

The nationwide incidence of sarcoma or TIM (2013–2016) was measured using the 2013 WHO classification and confirmed by a second independent review by expert pathologists. Simple clinical characteristics, yearly variations and correlation of incidence with published clinical trials are presented and analyzed.

**Results:**

Over 150 different histological subtypes are reported from the 25172 patients with sarcomas (n = 18712, 74,3%) or TIM (n = 6460, 25.7%), with n = 5838, n = 6153, n = 6654, and n = 6527 yearly cases from 2013 to 2016. Over these 4 years, the yearly incidence of sarcomas and TIM was therefore 70.7 and 24.4 respectively, with a combined incidence of 95.1/10^6^/year, higher than previously reported. GIST, liposarcoma, leiomyosarcomas, undifferentiated sarcomas represented 13%, 13%, 11% and 11% of tumors. Only GIST, as a single entity had a yearly incidence above 10/10^6^/year. There were respectively 30, 64 and 66 different histological subtypes of sarcomas or TIM with an incidence ranging from 10 to 1/10^6^, 1–0.1/10^6^, or < 0.1/10^6^/year respectively. The 2 latter incidence groups represented 21% of the patients with 130 histotypes. Published phase III and phase II clinical trials (p<10^−6^) are significantly higher with sarcomas subtypes with an incidence above 1/10^6^ per.

**Conclusions:**

This nationwide registry of sarcoma patients, with exhaustive histology review by sarcoma experts, shows that the incidence of sarcoma and TIM is higher than reported, and that tumors with a very low incidence (1<10^6^/year) are less likely to be included in clinical trials.

## Introduction

Sarcomas are a group of rare malignant diseases of the connective tissues, with heterogeneous clinical presentations and natural histories. The incidence of sarcoma was reported 15 years ago to be close to 2/10^5^/year, but more recently, the global incidence was reported to be higher, ranging from 3 to 7 /10^5^/year [1–16]. The incidence of sarcoma varies across countries and according to the date of the study [2–16] and is therefore not precisely known. Similarly, the incidence of each individual histological subtypes is unclear and sarcomas are misdiagnosed in up to 30% of cases [1, 5, 6, 11, 17]. These patients with misdiagnosed sarcomas may not be treated according to clinical practice guidelines [1–21]. Clinical practice guidelines recommend that the diagnosis of sarcoma should be confirmed by an expert pathologist and that the management of sarcoma patients should be performed by a dedicated multidisciplinary team, including expert pathologists and surgeons, treating a minimal number of patients per year [5–7]. Central pathology review of sarcoma cases was shown to be cost effective, reducing both morbidity, mortality and cost of management [22, 23].

Since 2010, the French National Cancer Institute (INCa) funded a pathology network (RRePS) and a clinical network (NETSARC) for sarcoma, to improve the quality of management of sarcoma patients. Subsequently, a dedicated bone sarcoma network named RESOS was created. Initially, the network composed of 23 expert reference centers for pathology (RRePS) was in charge of the histological review of each suspected case of sarcoma nationwide. Since 2019, all three networks have merged in a single network (acronym NETSARC+). The shared online database (see rreps.org and netsarc.org) gathers all cases of sarcoma reviewed by a multidisciplinary tumor board. It collects data related to diagnostic, therapeutic management, relapse and survival.

Since the January 1^st^ 2010, this database prospectively has included over 60000 patients with sarcoma and connective tissue tumor of intermediate malignancy (TIM), as defined according to the WHO 2013 classification [1]. Since 2013, the overall accrual in the database reached a plateau, providing a near exhaustive collection of cases in this country.

The global nationwide incidence of sarcoma has seldom been reported. Taking advantage of the organized reference center networks and expert pathology reviewing, we report here the incidence of the different histological subtypes of sarcomas and TIM from the NETSARC+ database from 2013 to 2016.

## Patient, material and methods

### The NETSARC+ network and the referral of the pathology samples to the network of experts

The RRePS (an acronym standing for Reseau de Relecture en Pathologie des Sarcomes, i.e. Network of Sarcoma Pathology Reviewing, gathering 23 centers), NetSarc (Network for Sarcoma, in charge of clinical management, gathering 26 centers), and ResOs (Network for Bone Sarcomas, dedicated to bone sarcoma pathology and clinical network) networks were merged into NETSARC+ (Network of Sarcomas Reference Centers) in 2019.

The organization of these networks has been previously reported [24, 25]: each RRePS and NetSarc centers hold a multidisciplinary tumor board gathering sarcoma specialized pathologist(s) (S1 Table), radiologist(s), surgeon(s), radiation oncologist(s), medical oncologist(s), molecular biologist(s), orthopedist(s) and pediatrician(s).

The missions given by the French National Cancer institute (INCa) funding the networks were, among others, to review all pathology samples and to collect a set of anonymized data to monitor patient outcome. Since 2010, it is mandatory for the primary pathologist to refer all suspected cases of sarcomas or TIM to one of these reference centers.

### The RRePS/NetSarc database

These databases have been approved by the French Ethic Committee and Agency in charge of non-interventional trials: Comité consultatif sur le traitement de l’information en matière de recherche dans le domaine de la Santé (CCTIRS: number of approval 09.594) and Commission Nationale Informatique et Liberté (CNIL: number of approval 909510). THe consent was obtained orally.

All sarcoma/TIM or suspected sarcoma/TIM patient cases discussed during the multidisciplinary tumor board (MDTB) of all 26 centers were recorded in the electronic online database, by a dedicated team of Clinical research assistant (CRAs), supervised by the Coordinating centers (Unicancer Comprehensive Cancer Centers: Centre Leon Bérard, Gustave Roussy, Institut Bergonié, as well as CHU Tours & CHU Nantes). Patients files may be presented at different stages of care process, before any diagnostic procedure, before initial biopsy, before primary surgery, after primary surgery, at relapse, and/or in case of a possible inclusion in a clinical trial as previously described [24, 25]. All patients had the option to opt out the initiave, or could orally consent to the pathology review of their biopsy, the registry in the national network database according to the national laws at that time, as well as benefit from the recommendations of the French National Cancer Institute.

The databases were therefore not generated from clinical trial data, and we built to monitor and improve the management of patients with sarcomas in France. According to the national legislation, the activity of the three networks did not have to be reviewed by the national ethics committees, compared to clinical studies (the Comité de Protection des Personnes). However, each local hospitals (i.e. the reference centers) used their internal procedure (internal institutional review board) to approve participation to this work. This approval was mandatory for the participation of the given center to any of the 3 networks.

Oral consent is documented through a standard information sheet given to all patients in each institution in the welcome leaflet. Patients are able to opt out, and this information is taken into account during multidisciplinary tumor board discussions of the cases.

Patients and treatment data were prospectively collected and regularly updated by the dedicated study coordinators. The cases were obtained directly from the pathologist laboratories, or referred to the expert pathology laboratory for diagnosis confirmation for a fraction of the patients who were first seen by the clinical MDT. This double source of entry contributed to improve the exhaustiveness of the collection of cases. Of note, the database includes on purpose a limited set of data, describing for example patients and tumor characteristics, surgery, relapse and survival [24, 25], centers performing the first resection, as well as potential secondary surgery types and sites, the final quality of resection.

The RRePS/NetSarc database may therefore give a nearly exhaustive representation of sarcoma cases to assess sarcoma incidence and prevalence in France.

The database is not systematically updated for follow-up by the CRA, as for clinical trial processes, but all baseline and first therapeutic information are completed until the end of the first line treatment. This includes all pathology reviews, which are therefore as presented, the final diagnoses, with a median follow-up of the series of 17 months in the recent publications of the same dataset (25). Importantly, since 2019, the data (survival and all treatments) from the nationwide database of the social security system (SNDS, https://www.snds.gouv.fr/SNDS/Accueil) is used to update the latest survival information of these patients as part of the Health DataHub Deepsarc project (https://www.health-data-hub.fr/outil-de-visualisation), now ensuring an exhaustive follow-up information.

Of note, the diagnosis of sarcomas of TIM (e.g. lipoma, carcinoma, lymphoma…) was not confirmed by the NetSarc MDTB for about 24% of the patients (not shown).

All data presented here were extracted from the common online database available online for a period of 4 years between 2013 and 2016. These 4 years were selected since: 1) the yearly incidence of sarcoma and TIM started to plateau since 2013, and 2) data monitoring and implementation is still ongoing since 2017.

### Presentation of the data

The 2013 WHO classification of sarcomas and connective tissue tumors was used from January 1^st^ 2013 to describe the histological subtypes in the database, taking advantage of the contribution of the 2013 version (since April 2012) by one of the senior authors (JMC) of the current article, [1]. Monthly physical meetings of the pathologist network to review complex cases have facilitated the homogeneity of data collection within this group. Both sarcomas and TIM were included in the database. Tumors of intermediate malignancy designates connective tissue tumors with the capacity to invade the surrounding tissues, with very rare metastases (1). These include for instance, aggressive fibromatosis, dermatofibrosarcoma protuberans, atypical lipomatous tumors …

The number of patients for each individual histological subtype of sarcoma or TIM per year, from 2013 to 2016, is therefore presented in the tables. To facilitate the comparison with other databases using previous classifications, the incidence of tumor groups (e.g. “uterine sarcomas”) are also presented in the tables. Each individual histotype, (e.g. WDLPS) and groups of histotypes are presented when clinically relevant (e.g. “WD and DDLPS”, or “liposarcoma”, “leiomyosarcomas”). Conversely, when a grouping did not exert relevance in clinical routine (e.g. “fibroblastic and myofibroblastic tumors” in Table 1), no new entity was described.

**Table 1:** Incidence of visceral and soft tissue sarcoma and tumors of intermediate malignancy over the 4 years of the study.

	**2013**	**2014**	**2015**	**2016**	**Total**	**Incidence**
						**/10**^**6**^**/year**
**Adipocytic tumours**	**744**	**821**	**817**	**865**	**3247**	**12,299**
Atypical lipomatous tumour /						
well-differentiated liposarcoma	289	304	314	357	1266	4,795
Liposarcoma–dedifferentiated	304	344	341	356	1345	5,095
**Myxoid Round Cell LPS**	**99**	**106**	**108**	**96**	**409**	**1,549**
Liposarcoma—myxoid	81	90	95	89	355	1,345
Liposarcoma—round cell	18	16	13	7	54	0,205
Liposarcoma—pleomorphic	31	41	36	31	139	0,527
Lipomatous spindle cell/pleomorphic	0	1	0	0	1	0,004
Liposarcoma NOS	21	25	17	22	85	0,322
Liposarcoma—mixed type	0	0	1	1	2	0,008
**Fibroblastic & myofibroblastic tumours**	**1041**	**1047**	**1115**	**1147**	**4349**	**16,473**
Desmoid fibromatosis	307	295	357	381	1340	5,072
Lipofibromatosis	3	0	5	0	8	0,030
Giant cell Fibroblastoma	2	4	4	1	11	0,042
Dermatofibrosarcoma Protuberans	261	270	258	251	1040	3,939
**Solitary fibrous tumour (all)**	**210**	**222**	**242**	**252**	**925**	**3,504**
• Solitary fibrous tumor	166	178	193	214	751	2,845
• High risk SFT	44	43	49	38	174	0,659
Inflammatory myofibroblastic Tum.	32	39	33	41	145	0,549
Low grade Myofibroblastic Sarc.	3	5	3	2	13	0,049
Myxoinflammatory Fibroblastic Sarc.	6	6	6	5	23	0,087
Infantile fibrosarcoma	3	2	1	4	10	0,038
Adult fibrosarcoma	11	4	9	4	28	0,106
Myxofibrosarcoma	162	160	152	156	630	2,386
Low grade fibromyxoid sarcoma	33	30	35	38	136	0,515
Sclerosing epithelioid fibrosarcoma	8	11	10	12	41	0,155
**So-called fibrohistiocytic tumours**	**29**	**16**	**37**	**24**	**106**	**0,402**
Intermediate fibrohistiocytic tumors (NOS)	0	0	2	3	5	0,019
Malignant tenosynovial giant cell tum.	1	0	0	1	2	0,008
Plexiform fibrohistiocytic tumors	7	7	9	6	29	0,110
Giant cell tumour of soft tissue	21	9	26	14	70	0,265
**Vascular tumours**	**398**	**377**	**381**	**364**	**1520**	**5,758**
Retiform hemangio-endothelioma	1	3	3	2	9	0,034
Papillary intralymphatic						
angioendothelioma	0	0	0	1	1	0,004
Composite hemangioendothelioma	1	1	1	0	3	0,011
Kaposi sarcoma	191	165	162	145	663	2,511
Kaposiform hemangioendothelioma	1	1	1	1	4	0,015
Pseudomyogenic hemangioendothelioma	1	3	0	2	6	0,023
Epithelioid hemangioEndothelioma	27	20	30	23	100	0,379
Angiosarcoma	176	183	182	187	728	2,758
Intermediate vascular tumours (NOS)	0	1	2	3	6	0,023
**Pericytic (perivascular) tumours**	**4**	**4**	**1**	**1**	**10**	**0,038**
Malignant glomus tumour						
**Smooth muscle (SM) tumours**	**646**	**698**	**669**	**666**	**2679**	**10,148**
SM tumor of undetermined malignancy	20	47	23	32	122	0,462
Metastatic leiomyoma	0	0	0	2	2	0,008
**Leiomyosarcoma (All)**	**626**	**651**	**646**	**632**	**2555**	**9,679**
Leiomyosarcoma (NOS)	247	263	287	297	1094	4,144
Leiomyosarcoma -differentiated	245	243	240	217	945	3,580
Leiomyosarcoma–poorly differentiated	134	145	119	118	516	1,955
**Skeletal muscle sarcoma (RMS)**	**145**	**157**	**173**	**133**	**608**	**2,303**
**Embryonal RMS**	**50**	**45**	**60**	**34**	**189**	**0,716**
• Embryonal RMS sarcoma—botryoid type	8	6	6	3	23	0,087
• Embryonal rhabdomyosarcoma usual type	35	31	47	24	137	0,519
• Embryonal rhabdomyosarcoma spindle cell	7	8	7	7	29	0,110
Alveolar RMS	27	36	35	25	123	0,466
Pleomorphic RMS	28	38	42	36	144	0,545
Sclerosing RMS	2	3	3	3	11	0,042
Spindle cell RMS	13	8	9	9	39	0,148
Adult spindle cell RMS	0	0	1	4	5	0,019
RMS NOS	21	25	23	19	88	0,333
Ectomesenchymoma: Mal. mesenchymoma	4	2	0	3	9	0,034
**Gastrointestinal stromal tumors (GIST).**	**736**	**792**	**913**	**831**	**3272**	**12,394**
**Chondro-osseous tumours**						
Extraskeletal osteosarcoma	**25**	**25**	**32**	**14**	**96**	**0,364**
**Peripheral nerve sheath tumours**	**75**	**68**	**69**	**74**	**286**	**1,083**
**MPNST (all)**	**72**	**64**	**62**	**66**	**264**	**1.000**
MPNST—epithelioid type	0	2	1	3	6	0,023
MPNST—usual type	36	7	14	28	85	0,322
Malignant peripheral nerve sheath tumour	36	55	47	35	173	0,655
Malignant Triton tumour	0	2	3	5	10	0,038
Malignant granular cell Tumour	3	2	4	0	9	0,034
Malignant perineurioma	0	0	0	3	3	0,011
	**2013**	**2014**	**2015**	**2016**	**Total**	**Incidence**
						**/10e6/year**
**Tumours of uncertain differentiation**						
Atypical fibroxanthoma	114	107	89	119	429	1,625
Angiomatoid fibrous histiocytoma	9	15	10	9	43	0,163
Ossifying fibromyxoid Tumour	7	7	5	13	32	0,121
**Myoepithelioma, myoepithelial carcinoma,**						
**& mixed tumour**	**31**	**26**	**18**	**18**	**93**	**0,353**
• Myoepithelioma	30	26	15	14	85	0,322
• Malignant myoepithelial Tumour	0	0	1	1	2	0,008
• Mixed tumour	1	0	2	3	6	0,023
Haemosiderotic fibrolipomatous tumour	0	2	0	7	9	0,034
Phosphaturic mesenchymal tumour	0	1	2	2	5	0,019
NTRK-rearranged spindle cell neoplasm (emerging) Not reported in NETSARC (so far)						
**Synovial sarcoma**	**103**	**101**	**133**	**105**	**442**	**1,674**
• Synovial sarcoma–NOS	23	18	29	21	91	0,345
• Synovial sarcoma—biphasic	11	19	23	17	70	0,265
• Synovial sarcoma–monophasic	60	57	67	60	244	0,924
• Synovial sarcoma—poorly Differentiated	9	7	14	7	37	0,140
**Epithelioid sarcoma (all)**	**29**	**30**	**28**	**33**	**120**	**0,455**
• Epithelioid sarcoma	23	28	25	22	98	0,371
• Undifferentiated epithelioid sarcoma	6	2	3	11	22	0,083
Alveolar soft part sarcoma	10	7	8	6	31	0,117
Clear cell sarcoma of soft tissue	13	16	26	16	71	0,269
Extraskeletal myxoid chondrosarcoma	15	12	20	11	58	0,220
Desmoplastic small round cell tumour	14	9	12	17	52	0,197
Extrarenal rhabdoid tumour	6	13	16	16	51	0,193
SMARCA4-deficient thoracic sarcoma	0	0	6	9	15	0,057
**PEComa, including angiomyolipoma**	**13**	**27**	**15**	**29**	**86**	**0,326**
• PECOMA—NOS	13	25	11	18	67	0,254
• Malignant PECOMA	0	2	4	13	19	0,072
Intimal sarcoma	14	12	11	9	46	0,174
**Undifferentiated sarcoma (all)**	**566**	**627**	**784**	**740**	**2717**	**10,292**
• Undifferentiated pleomorphic sarcoma	290	367	470	429	1556	5,894
• Undifferentiated sarcoma	110	87	154	79	430	1,629
• Undifferentiated sarcoma -NOS	125	130	111	57	423	1,602
• Undifferentiated spindle cell sarcoma	41	43	49	175	308	1,167
Low grade sinonasal sarcoma	2	0	0	3	5	0,019
Melanotic neuroectodermal tumour infancy	0	0	0	1	1	0,004
**Phyllode sarcoma**	**32**	**25**	**46**	**35**	**138**	**0,523**
**Sarcomas or TIM NOS**						
Sarcoma NOS	166	197	259	189	809	3,064
Tumors of intermediate malignancy (NOS)	7	15	13	17	52	0,197

In red, groups of tumors (e.g. adipocytic tumors) according to the WHO classification, in bold, subgroups of tumors considered relevant (e.g. “all leiomyosarcomas”, “All MPNST”). Note that in few of these patients with sarcomas and TIM mostly of soft tissue /visceral origin, the primary site was indicated as being from the bone. They are described specifically in Table 3. Phyllodes tumors refer to all malignant phyllodes.

To estimate the incidence of these tumors, we used the official number of French citizens from 2013 to 2016, which were respectively 65.56, 66.13, 66.42 and 66.60 million inhabitants.

### Matching histotypes with published clinical trials

For each individual histotype, we searched Pubmed to identify published dedicated clinical trials. The name of the histological entity (e.g. angiosarcoma, pleomorphic liposarcoma…) was filtered for clinical trial, adding « phase III », « randomized phase II », or « phase II ». Pubmed was interrogated between January 15^th^ 2020 and January 30^th^ 2020. For the presentation of these data, all sarcoma histotypes or groups of histotypes, were ranked according to the order of decreasing incidence. When at least one phase II (light blue), one randomized phase II (dark blue), or one phase III (green) was published in the literature this was indicated in the line of the histotype using this colour code. It was also also used for the 3 columns of sarcoma/TIM with decreasing incidences to facilitate the visibility of the correlation between incidence of sarcoma and availability of clinical trials on the figure.

### Statistical analyses

The number of patients per year with the different histotypes is presented in tables. To analyze the variation of incidence over the 4 years, a Poisson Regression was used. Six histotypes with a significant variation in the period of observation are graphically detailed by overlaying the observed incidence, a linear regression over time and a spline. The comparison of the frequency of published clinical trials per histological subtypes or groups of subtypes was performed using the chi square or Fisher’s exact test with a threshold p value of p<0.05. All statistical tests were two-sided. All statistical analyses were performed using SPSS (v 23.0) (IBM, Paris, France).

## Results

### Incidence of sarcoma and TIM in NetSarc

Tables 1–3 present the incidence of the individual histological subtypes of soft tissue sarcomas/TIM (Table 1), visceral sarcomas/TIM (Tables 1 and 3 for uterine sarcoma), bone sarcomas/TIM (Tables 2 and 3) included in the database (gathering the RRePS, NetSarc, and ResOs) from 2013 to 2016, a period where the data are expected to be close to exhaustive nationwide.

**Table 2:** Incidence of bone sarcoma over the 4 years of the study.

	2013	2014	2015	2016	Total	Incidence
						**/10**^**6**^**/year**
**Undifferentiated small round cell sarcomas**						
**(SRCS) of bone and soft tissue**						
Ewing sarcoma	151	163	153	147	614	2,326
SRCS with EWSR1-non-ETS fusions	6	6	8	36	56	0,212
CIC-rearranged sarcoma	1	3	3	4	11	0,042
BCOR-rearranged Sarcoma	2	0	2	3	7	0,027
**Bone tumours**						
**Chondrogenic tumours**						
Chondroblastoma	11	16	9	16	52	0,197
Chondromyxoid fibroma	4	7	12	3	26	0,098
**Chondrosarcoma (all)**	**227**	**211**	**244**	**285**	**967**	**3,663**
Central atypical cartilaginous						
tumour/chondrosarcoma, gd 1	2	10	19	45	76	0,288
Central chondroS grades 2 and 3	22	33	35	27	117	0,443
Chondrosarcoma NOS	164	125	143	140	572	2,167
Peripheral chondrosarcoma	5	6	8	20	39	0,148
Periosteal chondrosarcoma	8	4	6	7	25	0,095
Clear cell chondrosarcoma	4	3	2	5	14	0,053
Mesenchymal chondrosarcoma	3	7	11	10	31	0,117
Dedifferentiated chondrosarcoma	19	23	20	31	93	0,352
**Osteogenic tumors**						
Osteoblastoma	5	9	8	10	32	0,121
**Osteosarcoma (all)**	**330**	**362**	**370**	**376**	**1438**	**7,122**
Low grade central osteosarcoma	4	4	4	7	19	0,072
Low-grade central osteosarcoma	2	1	1	3	7	0,027
Dediff. low grade central osteosarcoma	2	3	3	4	12	0,045
Osteosarcoma	154	169	170	168	661	2,504
Osteosarcoma NOS	39	57	62	72	230	1,106
Conventional osteosarcoma	111	105	105	96	417	1,580
Osteoblastoma-like osteosarcoma	1	0	0	1	2	0,008
Telangiectasic osteosarcoma	2	7	2	5	16	0,061
Small cell osteosarcoma	1	0	1	2	4	0,015
**Parosteal osteosarcoma**	**7**	**7**	**16**	**10**	**40**	**0,152**
• Parosteal osteosarcoma	6	4	10	7	27	0,102
• Dedifferentiated parosteal osteoSarc	1	3	6	3	13	0,049
Periosteal osteosarcoma	1	4	0	0	5	0,019
High-grade surface osteosarcoma	6	5	6	8	25	0,095
**Fibrogenic tumors**						
Desmoplastic fibroma of bone	0	0	2	4	6	0,023
Fibrosarcoma of the bone	0	1	3	0	4	0,015
**Vascular tumor of bone**						
Epithelioid haemangioendothelioma	1	2	5	0	8	0,030
Angiosarcoma of bone	7	6	7	9	29	0,110
**Osteoclastic giant-cell rich**						
Aneurysmal bone cyst	14	9	22	8	53	0,201
Giant cell tumour of bone	76	88	87	67	318	1,204
Malignant/dedifferentiated GCTB	0	0	2	4	6	0,023
**Notochordal tumors**						
• Conventional chordoma	35	33	42	54	164	0,621
• Dedifferentiated chordoma	0	1	1	0	2	0,008
**Adamantinoma**	8	1	2	8	19	0,072
**Langerhans cell histiocytosis**	4	8	4	4	20	0,076

In red, groups of tumors (e.g. chondrogenic tumors) according to the WHO classification, in bold, subgroups of tumors considered relevant (e.g. all osteosarcoma).

**Table 3:** Incidence of uterine and rare bone sarcoma over the 4 years of the study.

	2013	2014	2015	2016	Total	Incidence
						**/10e6/year**
**Uterine sarcoma**	**242**	**311**	**285**	**300**	**1138**	**4,311**
**Endometrial stromal sarcoma, low grade (all)**	**57**	**64**	**55**	**62**	**238**	**0,902**
• Endometrial stromal nodule	2	1	5	8	16	0,061
• Endometrial stromal sarcoma	0	3	2	5	10	0,038
• Endometrial stromal sarcoma-low grade	55	60	48	49	212	0,803
Endometrial stromal sarcoma—high-grade	1	5	13	22	41	0,155
Adenosarcoma	28	35	42	51	156	0,591
Undifferentiated uterine sarcoma	37	49	38	17	141	0,534
Uterine tumour resembling ovarian sex cord	5	1	4	7	17	0,064
**Uterine leiomyosarcoma**						
(extracted from the LMS group above)	**114**	**157**	**133**	**141**	**545**	**2,064**
**Rare bone sarcomas**						
(extracted from the histological groups in Table 1)						
**All undifferentiated sarcoma of bone**	**36**	**31**	**38**	**47**	**152**	**0,576**
• Undifferentiated pleomorphic sarcoma	16	20	12	21	69	0,261
• Undifferentiated sarcoma	16	11	21	8	56	0,212
• Undifferentiated spindle cell sarcoma	4	0	5	17	26	0,098
• Undifferentiated epithelioid sarcoma	0	0	0	1	1	0,004
Leiomyosarcoma of bone	11	15	5	9	40	0,152
Synovial sarcoma of bone	4	2	2	1	9	0,034
Rhabdomyosarcoma of bone	2	2	1	4	9	0,034
BCOR Sarcoma of bone	1	0	2	3	6	0,023
Myoepithelioma of bone	1	1	1	1	4	0,015
Liposarcoma of bone	0	2	0	2	4	0,015
Other histological subtypes of bone sarcomas	33	42	46	50	171	0,648
**Genetic predisposition of soft tissue and bone or HIV**						
Enchondromatosis	5	2	6	4	17	0,064
Li Fraumeni syndrome	3	3	4	4	14	0,053
Retinoblastoma	1	0	3	1	5	0,019
Multiple osteochondroma	2	2	11	5	20	0,076
Neurofibromatosis	28	28	24	25	105	0,398
Rothmund-Thomson	0	1	0	0	1	0,004
HIV	4	12	10	6	32	0,121
Other immunosuppression	3	13	4	5	25	0,095

In red, groups of tumors (e.g. low grade endometrial stromal sarcoma). UTROSC were identified by the network experts and included in the database: though borderline for this group of diseases, we chose to insert them in this list.

From 2013 to 2016, a total of 25172 patients were included in the database, with n = 5838, n = 6153, n = 6654, and n = 6527 of new patients each year. Of note, respectively N = 4435 N = 4977 and N = 5550 patients were included in each year from 2010 to 2012 (not shown). The NetSarc database contains over 150 individual histological subtypes (i.e. a single histological entity such as monophasic synovial sarcoma, low grade surface osteosarcoma, atypical lipomatous tumor) or groups of sarcomas (e.g. liposarcoma, leiomyosarcoma, osteosarcoma, where the grouping of individual histological entity when clinically relevant) (Tables 1–). The grouping are described in S2 Table. Twelve additional histological subtypes of bone sarcomas (leiomyosarcomas, synovial etc) were also distinguished in this work and described in Table 3. These histotypes usually arising from soft tissue are also included in Table 1. Finally, Table 3 also presents the incidence of sarcomas diagnosed in patients with reported genetic predispositions, such as Li Fraumeni syndrome.

The official numbers of the French population are 65.56, 66.13, 66.42 and 66.60 million inhabitants in respectively 2013, 2014, 2015, 2016. The estimated incidences of sarcomas and tumors of intermediate malignancy from 2013 to 2016 were 89.05, 93.04, 100.18, and 98.00 per million inhabitants respectively. Over these 4 years, the estimated yearly incidence of sarcomas and TIM was therefore 95,1/10^6^/year. There were 18712 (74%) patients with sarcomas (incidence 70.7/10^e^/year) and 6460 (26%, 24.4/10^6^/year) patients with TIM. The observed overall incidence of sarcoma and TIMs is therefore higher that previously reported [1–15].

### Over 100-fold difference in incidence in different sarcoma histotypes

To complete Table 1 data, S1 Fig presents the individual histotypes and relevant groups of histotypes (e.g. liposarcoma, leiomyosarcoma, uterine sarcomas) by increasing incidence. GIST, liposarcoma, leiomyosarcomas, undifferentiated sarcomas represent 13%, 13%, 11% and 11% of all sarcomas (47% all together). Only gastrointestinal stromal tumors, if considered as a single entity, exceeded a yearly incidence above 10/10^6^/ year (S1 Fig). The other histological types of sarcomas with a yearly incidence above 10/10^6^/year are 1) all liposarcomas, 2) all smooth muscle tumors, 3) all undifferentiated sarcomas, and 4) all fibroblastic or myofibroblastic tumors lumped together. This latter group is not clinically homogenous and usually not considered as a specific entity in clinical trials or retrospective studies.

**Fig 1:**
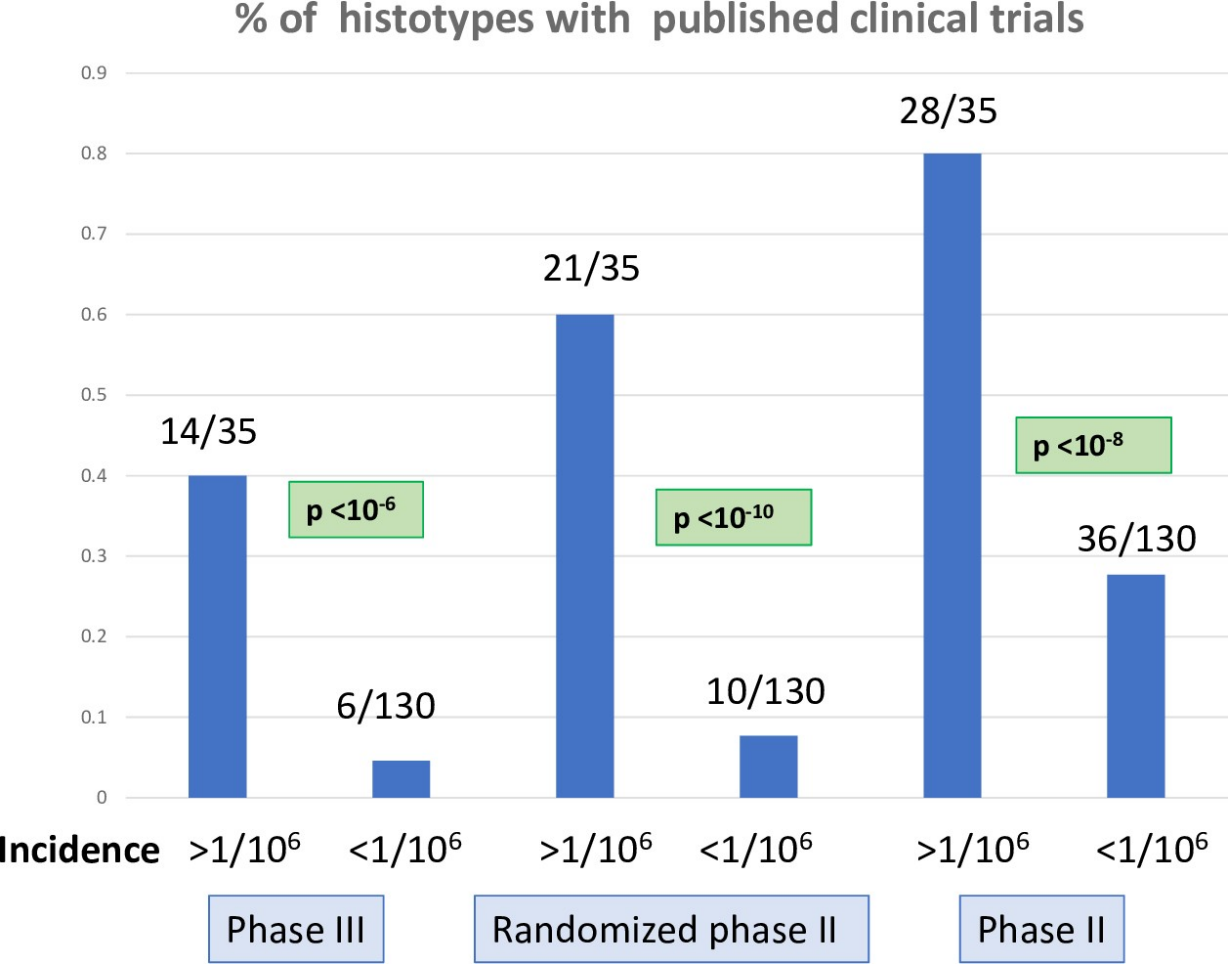
Published clinical trials in sarcoma and TIM histotypes. The histograms present the percentage of sarcoma histotypes and groups of histotypes with published clinical trials in Pubmed according to the incidence of the histotypes (>1/10^6^/year vs <1/10^6^/year). Numbers of histotypes with published phase III clinical trials (left), randomized phase II trials (center), and phase II trials (right) are indicated, with chi square p value for the comparison between the 2 incidence groups (>1/10^6^/year vs <1/10^6^/year). Histotypes were considered individually (e.g. monophasic synovial sarcoma) or globally (e.g. all synovial sarcoma).

Fig 1 presents the list of sarcomas or TIM with a decreasing incidence ranging from 10 to 1/10^6^/year, 1–0.1/10^6^ per year, or <0.1/10^6^/year. The 3 groups include respectively 30, 64 and 66 different histological subtypes or groups of histological subtypes. The groups with an incidence from 1–0.1/10^6^ per year, or <0.1/10^6^/year included respectively 4766 (19%) and 568 (2%) of the 25172 patients.

The mean age, sex ratio and sites for the different histotypes are presented in Table 4. It shows the large clinical heterogeneity of these tumors with a mean age ranging from 5 years (infantile fibrosarcoma) to 78 (atypical fibroxanthoma), and a sex ratio from 0 (for sexual organs) to 153 for adenosarcoma.

**Table 4:** Clinical characteristics of individual sarcoma histotypes.

Histotypes							Sites (%)*			
	Mean	F/M								
	Age	Ratio	GI	Gyn	H&N	I. trnk	L. limb	Trnk w	U. limb	Others
Adamantinoma	29,1	1,71	0,0	0,0	0,0	0,0	100,0	0,0	0,0	0,0
Adenosarcoma	61,3	51,00	1,9	89,7	0,0	3,8	0,6	0,6	0,0	3,2
Adult fibrosarcoma	69,5	1,15	0,0	7,1	7,1	10,7	17,9	28,6	17,9	10,7
Adult spindle cell rhabdomyosarcoma	52,4	0,25	0,0	0,0	20,0	0,0	20,0	40,0	20,0	0,0
Alveolar rhabdomyosarcoma	22,7	1,05	0,0	0,8	40,7	16,3	13,8	9,8	13,8	4,9
Alveolar soft part sarcoma	30,5	1,21	0,0	0,0	9,7	6,5	51,6	22,6	6,5	3,2
Aneurysmal bone cyst	30,2	1,12	0,0	0,0	3,8	1,9	39,6	32,1	20,8	1,9
Angiomatoid fibrous histiocytoma	27,1	0,87	0,0	0,0	7,0	11,6	37,2	18,6	25,6	0,0
Angiosarcoma	67,2	1,57	1,2	0,5	14,1	7,0	9,9	21,4	3,3	42,4
Atypical cartilaginous Tumour/ChondroS G1	45,0	1,38	0,0	0,0	3,9	0,0	36,8	14,5	43,4	1,3
Atypical fibroxanthoma	78,7	0,20	0,2	0,0	12,8	0,0	1,6	82,1	1,6	1,6
Atypical lipomatous tumor/WDLPS	64,2	0,84	0,9	0,1	2,7	29,8	44,3	14,9	5,8	1,4
BCOR sarcoma	18,9	0,40	0,0	0,0	0,0	28,6	28,6	42,9	0,0	0,0
Central chondrosarcoma	56,4	0,98	0,0	0,0	7,7	13,7	35,0	17,1	26,5	0,0
Chondroblastoma	24,2	0,44	0,0	0,0	5,8	0,0	63,5	17,3	13,5	0,0
Chondromyxoid fibroma	34,1	0,73	0,0	0,0	3,8	0,0	42,3	30,8	23,1	0,0
Chondrosarcoma NOS	54,1	0,91	0,3	0,3	13,5	20,6	25,5	21,3	17,0	1,4
Chordoma	61,7	0,71	0,0	0,0	13,4	0,6	0,0	85,4	0,0	0,6
CIC-DUX sarcoma	24,1	0,38	9,1	0,0	9,1	0,0	45,5	27,3	0,0	9,1
Clear cell chondrosarcoma	42,5	0,27	0,0	0,0	0,0	7,1	64,3	7,1	21,4	0,0
Clear cell sarcoma	42,3	0,87	12,7	0,0	5,6	2,8	46,5	14,1	14,1	4,2
Composite hemangioendothelioma	33,3	0,50	0,0	0,0	0,0	0,0	33,3	66,7	0,0	0,0
Conventional osteosarcoma	32,6	0,80	0,0	0,0	12,5	3,1	60,2	14,4	9,6	0,2
Dedifferentiated chondrosarcoma	64,0	0,94	0,0	0,0	1,1	11,8	48,4	30,1	7,5	1,1
Dedifferentiated chordoma	53,0	NA	0,0	0,0	0,0	0,0	0,0	100,0	0,0	0,0
Dedifferentiated low-grade central osteo	36,2	1,40	0,0	0,0	0,0	0,0	83,3	8,3	8,3	0,0
Dedifferentiated parosteal osteosarcoma	43,8	2,25	0,0	0,0	7,7	0,0	76,9	7,7	7,7	0,0
Dermatofibrosarcoma protuberans	45,0	1,02	0,0	0,1	3,0	0,5	19,7	61,3	13,8	1,5
Desmoid-type fibromatosis	43,8	2,17	15,9	0,1	3,3	7,7	7,4	59,0	6,3	0,4
Desmoplastic fibroma of bone	27,5	1,00	0,0	0,0	0,0	0,0	66,7	16,7	16,7	0,0
Desmoplastic round cell tumour	24,3	0,30	3,8	0,0	0,0	84,6	0,0	0,0	0,0	11,5
Embryonal rhabdomyosarcoma -botryoid type	10,7	2,29	0,0	43,5	21,7	0,0	0,0	0,0	0,0	34,8
Embryonal rhabdomyosarcoma—NOS	20,3	0,71	0,0	16,7	41,7	8,3	0,0	8,3	0,0	25,0
Embryonal rhabdomyosarcoma—spindle cell	19,0	0,45	0,0	6,9	31,0	37,9	0,0	6,9	3,4	13,8
Embryonal rhabdomyosarcoma—usual type	14,4	0,57	0,9	4,4	35,4	38,9	3,5	3,5	0,9	12,4
Endometrial stromal nodule	50,7	NA	0,0	81,3	0,0	0,0	0,0	18,8	0,0	0,0
Endometrial stromal sarcoma NOS	56,2	NA	0,0	80,0	0,0	20,0	0,0	0,0	0,0	0,0
Endometrial stromal sarcoma—high-grade	60,0	NA	0,0	95,1	0,0	4,9	0,0	0,0	0,0	0,0
Endometrial stromal sarcoma—low-grade	53,0	211,00	0,5	88,7	0,0	9,4	0,0	0,5	0,0	0,9
Epithelioid hemangioendothelioma	52,1	1,56	2,0	0,0	9,0	11,0	14,0	14,0	5,0	45,0
Epithelioid sarcoma	40,1	0,85	1,0	6,1	2,0	12,2	22,4	19,4	29,6	7,1
Ewing sarcoma	26,0	0,68	1,0	0,5	5,7	16,6	28,2	33,6	7,8	6,7
Extraskeletal myxoid chondrosarcoma	58,2	0,81	0,0	0,0	0,0	1,7	58,6	27,6	8,6	3,4
Extraskeletal osteosarcoma	63,1	0,78	0,0	1,0	1,0	1,0	44,8	24,0	19,8	8,3
Fibro-osseous tumour of bone NOS	36,0	NA	0,0	0,0	0,0	0,0	0,0	100,0	0,0	0,0
Fibrosarcomatous dermatofibrosarcoma prot.	45,6	0,65	0,0	0,0	3,4	1,7	18,8	65,0	10,3	0,9
Gastrointestinal stromal tumour (GIST),	65,4	0,94	94,8	0,1	0,0	4,8	0,0	0,2	0,0	0,1
Giant cell fibroblastoma	22,0	0,38	0,0	0,0	0,0	0,0	54,5	45,5	0,0	0,0
Giant cell tumour of bone	37,8	1,13	0,0	0,0	0,9	0,0	58,3	14,4	25,1	1,3
Giant cell tumour of soft tissues	47,5	1,41	0,0	0,0	7,1	1,4	47,1	8,6	35,7	0,0
Hemosiderotic fibrolipomatous tumour	45,4	3,50	0,0	0,0	0,0	0,0	100,0	0,0	0,0	0,0
High risk solitary fibrous tumour	64,4	0,85	2,9	0,6	8,0	31,0	6,3	13,8	1,7	35,6
High-grade surface osteosarcoma	44,6	0,92	0,0	0,0	24,0	12,0	44,0	20,0	0,0	0,0
Infantile fibrosarcoma	5,9	2,33	10,0	0,0	20,0	10,0	20,0	20,0	0,0	20,0
Inflammatory myofibroblastic tumour	39,3	1,10	11,0	4,1	11,7	54,5	6,2	6,9	5,5	0,0
Intermediate fibrohistiocytic tumours NOS	41,0	0,25	0,0	0,0	0,0	0,0	60,0	0,0	40,0	0,0
Intermediate vascular tumours NOS	64,7	5,00	0,0	0,0	16,7	0,0	0,0	83,3	0,0	0,0
Intimal sarcoma	58,9	0,92	0,0	0,0	0,0	39,1	2,2	0,0	0,0	58,7
Kaposi sarcoma	65,8	0,22	1,1	0,0	3,2	1,1	65,8	11,5	13,4	4,1
Kaposiform hemangioendothelioma	6	3,00	0,0	0,0	0,0	0,0	50,0	50,0	0,0	0,0
Langerhans cell histiocytosis	29,5	4,00	0,0	0,0	10,0	5,0	5,0	75,0	5,0	0,0
Leiomyosarcoma	63,5	2,18	4,8	35,3	7,1	18,7	15,1	8,0	4,9	5,9
Leiomyosarcoma—differentiated	63,1	1,23	4,9	18,2	6,1	24,2	18,4	11,5	9,7	6,9
Leiomyosarcoma—poorly-differentiated	70,3	0,73	3,7	8,9	29,7	9,7	19,4	15,3	7,6	5,8
Lipofibromatosis	10,3	1,00	12,5	0,0	12,5	0,0	12,5	50,0	12,5	0,0
Lipomatous spindle cell/pleomorphic tum	33,0	NA	0,0	0,0	0,0	0,0	100,0	0,0	0,0	0,0
Liposarcoma—dedifferentiated	67,9	0,60	2,0	0,3	1,6	69,5	11,4	9,5	2,2	3,5
Liposarcoma—mixed type	61,0	1,00	0,0	0,0	0,0	50,0	50,0	0,0	0,0	0,0
Liposarcoma—myxoid	47,8	0,81	0,3	0,3	0,0	4,8	77,5	14,4	2,3	0,6
Liposarcoma—NOS	64,2	0,57	1,2	1,2	2,4	38,8	31,8	11,8	8,2	4,7
Liposarcoma—pleomorphic	63,1	0,78	0,7	0,7	3,6	15,1	38,1	22,3	15,8	3,6
Liposarcoma—round cell	49,2	0,59	0,0	0,0	0,0	14,8	63,0	14,8	7,4	0,0
Low grade fibromyxoid sarcoma	42,5	0,97	0,0	0,0	9,6	5,9	34,6	36,8	11,0	2,2
Low grade myofibroblastic sarcoma	40,5	1,17	7,7	0,0	46,2	0,0	23,1	23,1	0,0	0,0
Low grade sinonasal sarcoma	37,6	1,50	0,0	20,0	80,0	0,0	0,0	0,0	0,0	0,0
Low-grade central osteosarcoma	33,6	2,50	0,0	0,0	0,0	14,3	71,4	0,0	14,3	0,0
Malignant glomus tumour	54,2	0,67	20,0	10,0	10,0	10,0	30,0	0,0	20,0	0,0
Malignant granular cell tumour	46,1	1,25	0,0	0,0	0,0	0,0	0,0	55,6	44,4	0,0
Malignant mesenchymoma	61,1	1,25	0,0	11,1	0,0	11,1	33,3	0,0	33,3	11,1
Malignant mixed tumor	67,5	NA	25,0	75,0	0,0	0,0	0,0	0,0	0,0	0,0
Malignant myoepithelial tumour	49,5	1,00	0,0	0,0	0,0	0,0	0,0	50,0	50,0	0,0
Malignant PECOMA	60,1	1,71	15,8	21,1	0,0	26,3	10,5	10,5	0,0	15,8
Malignant perineurioma	46,3	2,00	0,0	0,0	0,0	0,0	33,3	33,3	0,0	33,3
Malignant peripheral nerve sheath tumour	46,4	0,86	0,6	0,0	11,6	17,3	24,9	31,2	9,8	4,6
Malignant rhabdoid tumour	24,3	0,89	2,8	8,3	13,9	16,7	5,6	16,7	0,0	36,1
Malignant tenosynovial giant cell tumour	68,5	0,00	0,0	0,0	0,0	50,0	0,0	50,0	0,0	0,0
Malignant Triton tumour	34,7	1,00	0,0	0,0	30,0	40,0	0,0	30,0	0,0	0,0
Malignant/dedifferentiated giant cell tumor of the bone	40,0	1,00	0,0	0,0	0,0	0,0	80,0	0,0	20,0	0,0
Melanotic neuroectodermal tumour of infa	38,0	NA	0,0	0,0	0,0	100,0	0,0	0,0	0,0	0,0
Mesenchymal chondrosarcoma	34,9	0,72	0,0	0,0	29,0	12,9	29,0	25,8	0,0	3,2
Metastatic leiomyoma	39,0	NA	0,0	0,0	0,0	50,0	0,0	50,0	0,0	0,0
Mixed tumour	66,0	NA	0,0	0,0	0,0	50,0	50,0	0,0	0,0	0,0
MPNST—epithelioid type	43,2	2,00	0,0	0,0	0,0	16,7	16,7	50,0	16,7	0,0
MPNST—usual type	45,4	0,77	1,2	1,2	14,1	12,9	27,1	27,1	11,8	4,7
Myoepithelioma	50,6	0,89	0,0	0,0	3,5	3,5	37,6	27,1	25,9	2,4
Myxofibrosarcoma	68,9	0,70	0,2	0,0	2,7	2,1	46,0	18,6	28,4	2,1
Myxoinflammatory fibroblastic sarcoma	54,3	0,53	0,0	0,0	0,0	0,0	39,1	0,0	60,9	0,0
Osseous tumour rich in giant cell NOS	40,5	1,00	0,0	0,0	0,0	0,0	50,0	50,0	0,0	0,0
Ossifying fibromyxoid tumour	49,8	1,13	0,0	0,0	9,4	6,3	12,5	37,5	34,4	0,0
Osteoblastoma	26,5	0,48	0,0	0,0	3,2	0,0	19,4	58,1	19,4	0,0
Osteoblastoma-like osteosarcoma	29,0	NA	0,0	0,0	0,0	0,0	100,0	0,0	0,0	0,0
Osteogenic tumor of uncertain prognosis	22,0	NA	0,0	0,0	0,0	0,0	0,0	100,0	0,0	0,0
Osteosarcoma NOS	38,3	0,64	0,0	0,0	15,7	2,6	51,3	19,1	9,1	2,2
Papillary intralymphatic angioendothelioma	13,0	NA	0,0	0,0	0,0	0,0	0,0	100,0	0,0	0,0
Parosteal osteosarcoma	33,6	2,86	0,0	0,0	0,0	3,7	85,2	0,0	11,1	0,0
PECOMA—NOS	55,7	3,79	11,9	25,4	3,0	41,8	6,0	4,5	1,5	6,0
Periosteal chondrosarcoma	41,3	1,08	4,0	0,0	0,0	8,0	32,0	24,0	32,0	0,0
Periosteal osteosarcoma	19,8	4,00	0,0	0,0	0,0	20,0	80,0	0,0	0,0	0,0
Peripheral chondrosarcoma	37,3	0,63	0,0	0,0	0,0	10,3	33,3	33,3	23,1	0,0
Phosphaturic mesenchymal tumour	55,8	0,67	0,0	0,0	0,0	0,0	40,0	60,0	0,0	0,0
Phyllodes sarcoma	51,3	137,00	0,0	0,0	0,0	1,4	0,0	13,0	0,0	85,5
Pleomorphic rhabdomyosarcoma	67,0	0,58	1,4	7,6	5,6	11,8	36,1	19,4	11,1	6,9
Plexiform fibrohistiocytic tumour	23,1	1,23	0,0	0,0	6,9	3,4	34,5	24,1	31,0	0,0
Pseudomyogenic hemangioendothelioma	37,3	1,00	0,0	0,0	0,0	0,0	50,0	50,0	0,0	0,0
Retiform hemangioendothelioma	40,2	0,80	0,0	0,0	0,0	0,0	22,2	44,4	33,3	0,0
Rhabdomyosarcoma—NOS	41,7	0,80	1,1	13,6	17,0	23,9	11,4	4,5	5,7	22,7
Sclerosing epithelioid fibrosarcoma	55,8	1,05	0,0	0,0	9,8	17,1	14,6	41,5	9,8	7,3
Sclerosing rhabdomyosarcoma	45,2	0,38	0,0	0,0	9,1	0,0	72,7	0,0	9,1	9,1
Small cell osteosarcoma	21,8	0,33	0,0	0,0	0,0	25,0	25,0	0,0	25,0	25,0
SMARCA4-deficient thoracic sarcoma	48,3	0,25	13,3	0,0	0,0	40,0	0,0	0,0	0,0	46,7
Smooth muscle tumour of undetermined mal	50,6	4,30	6,6	59,8	0,8	15,6	5,7	7,4	4,1	0,0
Solitary fibrous tumour	58,0	1,25	1,2	1,1	15,4	21,0	13,3	18,6	4,3	25,0
Spindle cell rhabdomyosarcoma	38,8	0,63	5,1	2,6	17,9	20,5	28,2	10,3	12,8	2,6
Suspicion of giant cell tumour of bone	56,3	0,00	0,0	0,0	0,0	0,0	33,3	0,0	66,7	0,0
Sarcoma NOS	59,2	1,03	4,7	7,7	11,0	13,3	21,8	18,0	11,4	12,1
Synovial sarcoma—biphasic	41,2	0,84	1,4	0,0	5,7	5,7	52,9	17,1	10,0	7,1
Synovial sarcoma—monophasic	42,8	1,18	1,2	0,0	4,9	9,4	42,6	13,1	15,6	13,1
Synovial sarcoma NOS	45,6	1,22	0,0	1,1	5,5	8,8	34,1	13,2	16,5	20,9
Synovial sarcoma—poorly differentiated	45,2	0,85	0,0	0,0	5,4	10,8	29,7	18,9	8,1	27,0
Telangiectasic osteosarcoma	25,1	0,60	0,0	0,0	6,3	0,0	75,0	0,0	18,8	0,0
Tumour of intermediate malignancy NOS	47	1,14	5,8	3,8	13,5	13,5	21,2	19,2	21,2	1,9
Undifferentiated epithelioid sarcoma	70	0,47	0,0	0,0	13,6	18,2	22,7	13,6	27,3	4,5
Undifferentiated pleomorphic sarcoma	69,2	0,79	1,7	0,8	15,6	7,6	35,0	19,6	14,0	5,7
Undifferentiated round cell sarcoma	41,2	1,33	1,8	5,4	10,7	16,1	23,2	23,2	3,6	16,1
Undifferentiated sarcoma	66,7	0,89	1,4	3,3	15,8	13,0	29,5	21,4	8,8	6,7
Undifferentiated sarcoma—NOS	62,5	0,86	1,2	4,5	23,2	14,4	18,9	18,2	6,1	13,5
Undifferentiated spindle cell sarcoma	64,1	0,79	1,6	2,3	19,8	11,7	23,7	19,8	9,7	11,4
Undifferentiated uterine sarcoma	63,8	NA	0,0	96,1	0,0	2,9	0,0	1,0	0,0	0,0
Uterine tumour resembling ovarian sex co	48,1	NA	5,9	94,1	0,0	0,0	0,0	0,0	0,0	0,0

*: Sites: GI: gastrointestinal; Gyn: Gynaecological sites; H&N: head and neck; I. trnk: Internal trunk; L. Limb: lower limb; Trnk w: trunk wall; U. limb: upper limb.

Sarcoma histotypes are presented by alphabetical order. Age, sex ratio, and groups of sites are presented for each histotypes.

### Variable incidence of sarcoma histotypes over the 2013–2016 period

We investigated then the variability of the yearly incidence of these different tumors in the database. The analysis of variance of the observed incidence indicated a significant interaction between time and histology. S2 Fig presents the six histological subtypes with the mst significant variation between 2013 and 2016. Adenosarcoma, desmoid tumors, malignant pecoma, UPS, endometrial stromal sarcoma—high-grade increased over the 4-year period, while myoepithelioma showed a decrease of incidence (S2 Fig). The significance of these variations remains unclear and needs further investigation using comparable registries with a centralized review.

### Incidence of individual histotypes and published clinical trials

Table 1 and S1 Fig gives a graphic presentation in decreasing order of the incidence of the different histotypes and groups of histotypes. These were matched with published clinical trial data collected from Pubmed on a given histological group (e.g. liposarcoma) or specific histotype (e.g. pleomorphic liposarcoma). Phase III studies, randomized phase II studies and non-randomized phase II studies are indicated in green, dark blue and light blue respectively showing a variable access to clinical trial according to the incidence of the histotype. An histological subtype is considered “covered” by a trial only if the trial design contains a specific arm (phase II) or a specific strata (phase III) for a given histotype.

As expected, phase III trials are made available mostly for histotypes or groups of histotypes with an incidence >1/10^6^ per year (Fig 1). 14 of 35 (40%) histotypes and groups of histotypes with an incidence >1/10^6^ had a dedicated phase III study vs 6 of 130 (4.6%) histotypes for sarcomas with an incidence <1/10^6^ (p<10^−6^). 20100 (79,7%) patients of the database had a histotype for which no phase III trial had been reported. Twenty-one of 35 (60%) histotypes with an incidence >1/10^6^ had a dedicated randomized phase II study vs 10 of 130 (7.7%) histotypes for sarcomas with a incidence <1/10^6^ (p<10^−10^). 13154 (52.1%) patients of the database had a specific histotype for which no randomized phase II trial had been reported. Twenty-eight of 35 (80%) histotypes with an incidence >1/10^6^ had a dedicated phase II study vs 36 of 130 (27.9%) histotypes for sarcomas with an incidence <1/10^6^ (p<10^−8^). 6516 (25.8%) patients of the database had a specific histotype for which no phase II trial had been reported.

## Discussion

The objective of this work was to measure the incidence of individual histological subtypes of sarcomas and TIM according to the 2013 WHO classification. These cases were collected from the NETSARC+ database, combining the previous RRePS, ResOs and NetSarc databases. This work, supported by the French NCI, allowed to measure the incidence of sarcomas and TIM in a nationwide level. The mandatory central pathology review, in place since 2010, has facilitated the constitution of a close to exhaustive nationwide collection of patients with sarcoma and TIM. Since 2013, the number of patients included in the database per year is relatively stable suggesting that the database may indeed be close to exhaustiveness. We stopped the description in 2016, since the period spanning from 2017 to 2019 is still being monitored and updated by the CRAs of NETSARC+.

The first important observation is that the incidence of these tumors is higher than previously reported in each of these 4 years [1–15]. Recently published data from countries in 4 different continents reported an overall incidence ranging from 3 to 7.7/10^6^/year. The results of these studies are heterogeneous in terms of proportion distribution of the histotypes, ranging from 4 to 20% for undifferentiated sarcomas for instance. These observations suggest that mandatory central pathology review, implemented during this period, enabled to collect exhaustive data to assess the incidence of almost all subtypes. In addition, it relied on expert reviewing and therefore a more accurate diagnosis.

Importantly, there are no national registries for any cancers, including sarcomas in France and there are several limitations for using regional registries for the model of rare cancers. These limitations are 1) the already mentioned importance of centralized expert pathology review in sarcoma, which corrects the first diagnosis in >20% of the cases [5–7, 16], and 2) the lack of exhaustiveness of these registries for sarcomas; an ongoing collaborative work between NetSarc and the network of Regional registries (unpublished) indicates that the incidence of sarcoma and TIM is less than 70% of that observed in the present report. An increased collaboration is strongly needed between these structures.

Mandatory central pathology review is essential for these rare cancers and it can be achieved through several pathways: 1) each pathology review report indicates the mandatory review process, the sites as well as the contact information of the expert pathologists; all French pathologists are addressed this report multiple times per year; 2) when missed, patients presented in clinical MDT without pathology review are immediately referred to the closest expert pathology center; 3) the patients themselves exerted on multiple occasions their request for a pathology review, asking for a wide dissemination on the internet of the existence of networks of excellence and the mandatory pathology and clinical review process.

While the incidence of sarcoma and TIM observed here is higher than previously reported [1–15], it is important to point out that comparison with historical series has important limitations: some individual entities described here may have been described only very recently (e.g. GIST before 1999, solitary fibrous tumors, etc…). The numbers presented are those measured with a more recent classification which has dramatically evolved in recent decades.

The present work also confirms that sarcoma and TIM are a highly fragmented group of diseases. Over 150 different histotypes or groups of histotypes relevant for clinical practice are listed. The precise definition of this number was somehow challenging, and in part subjective for the selection of a relevant group of histotypes treated homogenously in routine in clinical trials (e.g. “liposarcoma”). It must be noted also that the actual number of different disease entities may actually be larger. GIST, for instance, gathers tumors with completely different genetic somatic alterations (of *KIT*, *PDGFRA*, *SDH*, *NF1*, *BRAF*, *NTRK3*) each with different natural histories.

The individual incidence of different sarcoma histotypes ranged from 10/10^6^ to less than 0.01/10^6^, i.e. a >1000-fold difference in incidence. Altogether sarcomas are considered as rare cancer, but the majority of individual subtypes are actually extremely rare. This is of course challenging for the understanding of the natural history of each individual subtypes, for the development of clinical trials, new treatments as well as defining standards of care.

Sarcoma and TIM are also a highly heterogeneous group of disease for clinical presentations as shown by the diversity of sex ratio and mean age of diagnosis. Each of these entities should therefore benefit from a specific research program to describe their natural history as well as the impact of current treatment on their disease course. This requires a coordinated effort, worldwide, to achieve this goal given the rarity of certain histotypes. This is currently being conducted by intergroup studies, and international networks such as WSN or more recently EURACAN [26–28]. This work also confirms the importance of national registries to investigate these rare subtypes.

An intriguing observation is the variation of incidence for these tumors over time, and statistically significant for several histotypes. There is however no clear explanation for this observation. Given the stable position of pathology experts involved in the network, this may not likely result from the variable pathology review. Epidemiological studies in other countries may be useful to confirm these variations, which may guide research on etiology of these rare sarcomas and TIM.

Another observation, expected by clinicians, is the link between the incidence and the availability of published prospective clinical research work to guide the management of individual subtypes. For decades, the medical treatment of sarcomas used a one-size-fits-all approach for all histotypes from phase II to III clinical trials, in particular in adults. For 15 years, histotype specific randomized phase II, III and phase II studies were implemented, starting with GIST. This is more the exception of the rule though. The majority of histotypes described in this work, especially those with an incidence under 1/10^6^/year have not had a dedicated phase II, randomized phase II or phase III clinical trial to adapt or guide clinical practice guidelines. This is graphically obvious, even though the mode of presentation of the figure amplified this phenomenon, since we presented both individual subtypes (e.g. monophasic synovial sarcoma, with no dedicated clinical trials to our knowledge) and pooled histotypes (e.g. all synovial sarcomas, where clinical trials were listed). It must be noted, however, that in the group with an incidence <0.1/10^6^/year, some histotypes were included in dedicated clinical trials. Due to the rarity of these tumors, clinical trials are expected to only be feasible at a global worldwide level, and randomization will not be feasible.

Overall, this calls for a revision of the criteria to define standard treatment for such rare tumors where phase III are hardly or not feasible [26–29]. Health authorities and reimbursement bodies should adapt their decisions on approval and reimbursement on the feasible level of evidence which could be reached for tumors with and incidence <1/10^6^ per year in order not to discriminate against patients with rare cancers. It is important to remember that altogether patients with rare cancers represent 22% of all patients with cancers, and about 30% of the mortality due to cancer [29].

This study has many limitations. We cannot exclude that patients may not reach our network despite the administrative incentive. This is true in particular for bone sarcoma and TIM (e.g. chondroblastomas, osteoblastoma, aneurysmatic bone cyst, etc.) which were collected more recently.

The work must also adapt to the rapidly evolving classification of sarcomas, including new molecular sub-classifications, which are not described here (for instance GIST, the novel NTRK sarcoma subgroup, BCOR, CIC-DUX4 sarcomas). To further explore the exhaustivity of the NETSARC+ database, an ongoing project connects it to the social security data base (SNDS) (the Health DataHub Deepsarc project), the single payer in France covering all citizens, across all diseases. This should enable to enhance the accuracy of these numbers.

In conclusion, this nationwide registry reports a higher than previously reported incidence of sarcoma and TIM at a nationwide level, over a 4-year period, with a central sarcoma pathologist expertise review. Our data provide a benchmark to be compared with other worldwide registries and confirm the limitations of clinical research in sarcomas with an incidence inferior to 1/10^6^ per year. The observation of variable incidence for specific histological subtypes is intriguing and requires further investigation by using data from other countries. Geographical research on the distribution of these cases over the national territory are currently ongoing.

## Supporting information

S1 Table Contributing pathologists of all centers RRePS & RESOS. This table includes per alphabetical order of institutions, the different pathologists contributing to this work. Note that a single reference center from RREPS or now NETSARC+ may include more than one institution.
(DOCX)

S2 Table Codes of the histotypes according to WHO 2013. (DOCX)

S3 Table Grouping of histotypes presented in the tables. (DOCX)

S1 Fig Published clinical trials in sarcoma and TIM histotypes. Tabular presentation of different sarcoma histotypes and groups of histotypes by decreasing order together with the documented published clinical trials in Pubmed. If phase III clinical trials are published, the box is highlighted in light green, if randomized phase II trials are published the box is highlighted in dark blue, if uncontrolled phase II trials are published the box is highlighted in light blue. Histotypes were considered individually (e.g. monophasic synovial sarcoma) or globally (e.g. all synovial sarcoma).
(TIF)

S2 Fig Variable incidence of sarcoma subtypes from 2013 to 2016. Presentation of the yearly variation of six different histotypes with significantly variable incidence in the period of observation.
(TIF)
