# Supplementary material for: Nationwide incidence of sarcomas and connective tissue tumors of intermediate malignancy over four years using an expert pathology review network
Source: PLoS One. 2021 Feb 25;16(2):e0246958. doi: 10.1371/journal.pone.0246958 (PMC7906477; doi:10.1371/journal.pone.0246958)
Supplement: S1 Table — This table includes per alphabetical order of institutions, the different pathologists contributing to this work. Note that a single reference center from RREPS or now NETSARC+ may include more than one institution. (DOCX) [file pone.0246958.s001.docx]

**S1 Table. Contributing pathologists of all centers RRePS & RESOS.**

Ambroise Paré APHP Jean-François EMILE

Ambroise Paré APHP Ute ZIMMERMANN

Angers ICO Isabelle VALO

APHP Bichat Francine WALKER

APHP Saint-Antoine Jean-François FLEJOU

APHP Saint-Antoine Pascale CERVERA

Bergonié Jean Michel COINDRE

Bergonié Agnès NEUVILLE

Bergonié Sophie LE GUELLEC

Bergonié Sabrina CROCE

Bergonié Marie KARANIAN

Bergonié Francois LE LOARER

Bergonié Gaetan MACGROGAN

Cabinet d’Amiens Pascal RICHARD

Cabinet de la Roquette Sara LAURENT-ROUSSEL

Cabinet de la Roquett e Philippe GROS

Cabinet Mathurin Isabelle et Françoise MOULONGUET PLANTIER)

Caen CFB Céline BAZILLE

Caen CFB Jean-Jacques MICHELS

CHR ORLEANS Flore DELALANDE

CHR ORLEANS Patrick MICHENET

CHRU Angers Anne CROUE

CHRU Brest Laurent DOUCET

CHRU Brest Isabelle QUINTIN-ROUÉ

CHRU Lille Sébastien AUBERT

CHRU Lille Xavier LEROY

CHRU Montpellier Christophe DELFOUR

CHRU Montpellier Jeanne RAMOS

CHRU Montpellier Eric FROUIN

CHU Nancy Béatrice MARIE

CHU Nancy Jean-Michel VIGNAUD

CHU Nancy Agnès LEROUX

CJP Florence MISHELLANY

Cochin Frédérique LAROUSSERIE

Cochin Virginie AUDARD

Cochin Marie-Cécile VACHER-LAVENU

CPS Strasbourg Jean-Pierre GHNASSIA

Curie Marick LAE

Curie Martine TRASSARD

Curie Jean-Marc GUINEBRETIÈRE

Cy-Path Anne-Valérie DECOUVELAERE

Dijon CGFL Francoise COLLIN

Dijon CGFL Céline CHARON BARRA

GHSN - LYON Mojgan DEVOUASSOUX

Haut-Lévèque Bordeaux Marion MARTY

Haut-Lévèque Bordeaux Marie-Laure JULLIE

Hôp. Charles Nicolle Emilie ANGOT

Hôp. Civil Strasbourg Mona MITCOV

Hôp. Nord Marseille Colette CHARPIN TARANGER

Hôp. Pontchaillou Nathalie STOCK

Hôp. Pontchaillou Florence BURTIN

IHP Nantes Geneviève AILLET

IHP Nantes Hélène CHOMARAT

IUCT Toulouse Sophie LE GUELLEC

IUCT Toulouse Anne GOMEZ-BROUCHET

IUCT Toulouse Philippe ROCHAIX

IUCT Toulouse Eliane MERY

IUCT Toulouse

Labo. des Feuillants Joelle REYRE

Lille COL Yves-Marie ROBIN

Limoges Dupuytren Isabelle POMMEPUY

Limoges Dupuytren Nicolas WEINBRECK

Lyon CLB Dominique RANCHERE

Lyon CLB Anne-Valérie DECOUVELAERE

Lyon CLB Marie KARANIAN

Lyon CLB Lyon Edouard Herriot Jean-Yves SCOAZEC

Marseille IPC Lénaïg MESCAM-MANCINI

Marseille IPC Bruno CHETAILLE

Marseille IPC Geneviève MONGES

Marseille IPC Flora POIZAT

MEDIPATH Nicolas WEINBRECK

MEDIPATH Bruno CHETAILLE

Montpellier ICM Marie Christine CHATEAU

Montpellier ICM Aurélie MARAN

Nancy ICL Agnès LEROUX

Nantes CHU Anne MOREAU

Nantes CHU Elisabeth CASSAGNAU

Nantes CHU Marie-Françoise HEYMANN

Nice CAL Isabelle BIRTWISLE-PEYROTTES

Paris Henri Mondor Nicolas ORTONNE

Pitié Salpétrière Catherine GENESTIE

Purpan Toulouse Janick SELVES

Rangueil Toulouse Anne GOMEZ-BROUCHET

Rennes CEM Nathalie STOCK

Saint-Louis Maxime BATTISTELLA

Strasbourg CHU Luc MARCELLIN

Strasbourg CHU Noëlle WEINGERTNER

Timone Corinne BOUVIER

Timone Nicolas MACAGNO

Tours Trousseau Gonzague DE PINIEUX

Tours Trousseau Anne DE MURET

Tours Trousseau Mélanie RAYEZ

Villejuif IGR Philippe TERRIER

Villejuif IGR Catherine GENESTIE
