# Supplementary material for: Nationwide incidence of sarcomas and connective tissue tumors of intermediate malignancy over four years using an expert pathology review network
Source: PLoS One. 2021 Feb 25;16(2):e0246958. doi: 10.1371/journal.pone.0246958 (PMC7906477; doi:10.1371/journal.pone.0246958)
Supplement: S2 Table — (DOCX) [file pone.0246958.s002.docx]

**S2 Table. Codes of the histotypes according to WHO 2013.**

**______________________________________________________________**

| **Histotypes** | **Codes** |
| --- | --- |
| **Soft tissue tumours (+visceral & bone)** |  |
|  |  |
| **Adipocytic tumours** |  |
|  |  |
| **Atypical lipomatous tumour / well-differentiated liposarcoma** |  |
| Atypical lipomatous tumour | 8850/1 |
| Liposarcoma – well differentiated | 8850/3 |
| Liposarcoma – dedifferentiated | 8858/3 |
| Myxoid liposarcoma | 8852/3 |
| Liposarcoma - myxoid | 8852/3 |
| Liposarcoma - round cell | 8852/3 |
| Liposarcoma - pleomorphic | 8854/3 |
| Myxoid pleomorphic liposarcoma (no data) | 8850/3 |
| Atypical spindle cell / pleomorphic lipomatous tumour | 8850/3 |
| Lipomatous spindle cell/pleomorphic tum | 8850/3 |
| Liposarcoma NOS | 8850/3 |
| Liposarcoma - mixed type | 8850/3 |
|  |  |
| **Fibroblastic and myofibroblastic tumours** |  |
| Desmoid fibromatosis | 8821/1 |
| Lipofibromatosis | 8851/1 |
| Giant cell Fibroblastoma | 8834/1 |
| Dermatofibrosarcoma Protuberans | 8832/1 |
| Solitary fibrous tumour | 8815/1 |
| High risk SFT | 8815/3 |
| Inflammatory myofibroblastic Tumour | 8825/1 |
| LG Myofibroblastic Sarcoma | 8825/3 |
| Myxoinflammatory Fibroblastic S | 8811/1 |
| Infantile fibrosarcoma | 8814/3 |
| Adult fibrosarcoma | 8810/3 |
| Myxofibrosarcoma | 8811/3 |
| Low grade fibromyxoid sarcoma | 8840/3 |
| Sclerosing epithelioid fibrosarcoma | 8840/3 |
|  |  |
| **So-called fibrohistiocytic tumours** |  |
| Intermediate fibrohistiocytic tumors | 9251/1 |
| Malignant tenosynovial giant cell tumors | 9252/3 |
| **Plexiform fibrohistiocytic tumors** | 8835/1 |
| Giant cell tumour of soft tissue | 9251/1 |
|  |  |
| **Vascular tumours** |  |
| Retiform hemangio-endothelioma | 9136/1 |
| Papillary intralymphatic angioendothelioma | 9135/1 |
| Composite hemangioendothelioma | 9136/1 |
| Kaposi sarcoma | 9140/3 |
| Kaposiform hemangioendothelioma | 9130/1 |
| Pseudomyogenic hemangioendothelioma | 9136/1 |
| Epithelioid hemangioEndothelioma | 9133/3 |
| Angiosarcoma | 9140/3 |
| Intermediate vascular tumours | 9136/1 |
|  |  |
| **Pericytic (perivascular) tumours** |  |
| Malignant glomus tumour | 8711/3 |
|  |  |
| **Smooth muscle tumours** |  |
| Smooth muscle tumour of undetermined malignancy | 8897/1 |
| Metastatic leiomyoma | 8897/1 |
| Leiomyosarcoma | 8890/3 |
| Leiomyosarcoma -differentiated | 8890/3 |
| Leiomyosarcoma – poorly differentiated | 8890/3 |
|  |  |
| **1.7. Skeletal muscle sarcoma (RMS)** |  |
| **1.7.1 Embryonal RMS** |  |
| Embryonal RMS sarcoma - botryoid type | 8910/3 |
| Embryonal rhabdomyosarcoma NOS | 8910/3 |
| Embryonal rhabdomyosarcoma usual type | 8910/3 |
| Embryonal rhabdomyosarcoma spindle cell | 8910/3 |
| **1.7.2. Alveolar RMS** | **8920/3** |
| **1.7.3. Pleomorphic RMS** | 8901/3 |
| **1.7.4. Sclerosing RMS** | 8912/3 |
| **1.7.5. Spindle cell RMS** | 8912/3 |
| **1.7.6. Adult spindle cell RMS** | 8912/3 |
| **1.7.7-8. RMS NOS** | 8912/3 |
| **1.7.9 Ectomesenchymoma : Malignant mesenchymoma** | 8921/3 |
|  |  |
| **1.8. Gastrointestinal stromal tumors (GIST).** | 8936/3 |
|  |  |
| **1.9:Chondro-osseous tumours** |  |
| Extraskeletal osteosarcoma | 9180/3 |
|  |  |
| **1.10:Peripheral nerve sheath tumours** |  |
| MPNST - epithelioid type | 9542/3 |
| MPNST - usual type | 9540/3 |
| Malignant peripheral nerve sheath tumour | 9540/3 |
| Malignant Triton tumour | 9561/3 |
| Malignant granular cell Tumour | 9580/3 |
| Malignant perineurioma | 8571/3 |
|  |  |
| **Tumours of uncertain differentiation** |  |
| Deep (aggressive) angiomyxoma : not reported | 8841/0 |
| Atypical fibroxanthoma | 8830/1 |
| Angiomatoid fibrous histiocytoma | 8836/1 |
| Ossifying fibromyxoid Tumour | 8842/3 |
| Myoepithelioma, myoepithelial carcinoma, and mixed tumour | 8982/3 |
| Myoepithelioma | 8982/0 |
| Malignant myoepithelial Tumour | 8982/3 |
| Mixed tumour | 8940/3 |
| Pleomorphic hyalinizing angiectatic tumour of soft parts | 8802/1 |
| Haemosiderotic fibrolipomatous tumour | 8811/1 |
| Phosphaturic mesenchymal tumour | 8990/3 |
| NTRK-rearranged spindle cell neoplasm (emerging) |  |
| Synovial sarcoma | 9040/3 |
| Synovial sarcoma - NOS | 9040/3 |
| Synovial sarcoma - biphasic | 9043/3 |
| Synovial sarcoma - monophasic | 9040/3 |
| Synovial sarcoma - poorly Differentiated | 9043/3 |
| Epithelioid sarcoma | 8804/3 |
| Undifferentiated epithelioid sarcoma | 8804/3 |
| Alveolar soft part sarcoma | 9581/3 |
| Clear cell sarcoma of soft tissue | 9044/3 |
| Extraskeletal myxoid chondrosarcoma | 9231/3 |
| Desmoplastic small round cell tumour | 8806/3 |
| Extrarenal rhabdoid tumour | 8063/3 |
| Malignant rhabdoid tumor | 8963/3 |
| SMARCA4-deficient thoracic sarcoma |  |
| PEComa, including angiomyolipoma |  |
| PECOMA - NOS | 8714/0 |
| Malignant PECOMA | 8714/3 |
| Intimal sarcoma | 9137/3 |
|  |  |
| **Undifferentiated sarcoma** |  |
| Undifferentiated pleomorphic sarcoma | 8802/3 |
| Undifferentiated sarcoma | 8803/3 |
| Undifferentiated sarcoma -NOS | 8804/3 |
| Undifferentiated spindle cell sarcoma | 8801/3 |
| Low grade sinonasal sarcoma |  |
| Melanotic neuroectodermal tumour of infancy |  |
|  |  |
| **Uterine sarcoma** |  |
|  |  |
| Endometrial stromal sarcoma, low grade | 8931/3 |
| Endometrial stromal sarcoma - high-grade | 8930/3 |
| Adenosarcoma | 8933/3 |
| Undifferentiated uterine sarcoma | 8805/3 |
| Uterine tumour resembling ovarian sex cord |  |
| Uterine leiomyosarcoma (extracted from the LMS group above) | 8890/3 |
|  |  |
| **Phyllode sarcoma** |  |
| **Sarcomas or TIM NOS** |  |
| Sarcoma NOS |  |
| Tumors of intermediate malignancy ALL |  |
|  |  |
| **Undifferentiated small round cell sarcomas of bone and soft tissue** | |
|  |  |
| **Ewing sarcoma** | 9364/3 |
| **Round cell sarcoma with EWSR1-non-ETS fusions** | 8803/3 |
| **CIC-rearranged sarcoma** | 8803/3 |
| **Sarcoma with BCOR genetic alterations** | 8803/3 |
|  |  |
| **Bone tumours** |  |
|  |  |
| **Chondrogenic tumours** |  |
| Chondroblastoma | 9230/1 |
| Chondromyxoid fibroma | 9241/0 |
| Osteochondromyxoma | 9211/0 |
| Synovial chondromatosis | 9220/0 |
| ***Chondrosarcomas*** |  |
| Central atypical cartilaginous tumour / chondrosarcoma, grade 1 |  |
| Cartilaginous tumour of uncertain prognosis | 9222/1 |
| Atypical cartilaginous Tumour/ Chondrosarcoma lowgrade | 9222/1 |
| Secondary peripheral atypical cartilaginous tumour / chondrosarcoma, grade 1 | 9222/1 |
| Central chondrosarcoma, grades 2 and 3 | 9220/3 |
| Chondrosarcoma | 9220/3 |
| Chondrosarcoma NOS | 9220/3 |
| Secondary peripheral chondrosarcoma | 9220/3 |
| Peripheral chondrosarcoma | 9220/3 |
| Periosteal chondrosarcoma | 9222/1 |
| Clear cell chondrosarcoma | 9242/3 |
| Mesenchymal chondrosarcoma | 8240/3 |
| Dedifferentiated chondrosarcoma | 9243/3 |
|  |  |
| **Osteogenic tumors** |  |
| Osteoblastoma | 9200/0 |
| Low grade central osteosarcoma | 9187/3 |
| Low-grade central osteosarcoma | 9187/3 |
| Dedifferentiated low grade central osteosarcoma | 9187/3 |
| ***Osteosarcoma*** | 9180/3 |
| Osteosarcoma NOS | 9180/3 |
| Conventional osteosarcoma | 9180/3 |
| Osteoblastoma-like osteosarcoma | 9180/3 |
| Telangiectasic osteosarcoma | 9183/3 |
| Small cell osteosarcoma | 9185/3 |
| Parosteal osteosarcoma | 9192/3 |
| Parosteal osteosarcoma | 9192/3 |
| Dedifferentiated parosteal osteosarcoma | 9192/3 |
| Periosteal osteosarcoma | 9193/3 |
| Periosteal osteosarcoma | 9193/3 |
| High-grade surface osteosarcoma | 9194/3 |
| Secondary osteosarcoma : not reported in NETSARC |  |
|  |  |
| **Fibrogenic tumors** |  |
| Desmoplastic fibroma of bone | 8823/1 |
| Fibrosarcoma of the bone | 8810/3 |
|  |  |
| ***Vascular tumor of bone*** |  |
| Hemangioma of bone: not reported in NETSARC |  |
| Epithelioid hemangioma of bone : not reported in NETSARC |  |
| Epithelioid haemangioendothelioma of bone ; | 9133/3 |
| Angiosarcoma of bone | 9120/3 |
|  |  |
| ***Osteoclastic giant-cell rich*** |  |
| Aneurysmal bone cyst | 9260/0 |
| Giant-cell tumor of bone | 9250/1 |
| Malignant/dedifferentiated GCTB | 9250/3 |
|  |  |
| ***Notochordal tumours*** |  |
| Benign notochordal cell tumor: not reportedin NETSARC |  |
| Conventional chordoma | 9370/3 |
| Dedifferentiated chordoma | 9370/3 |
|  |  |
| ***Other mesenchymal tumors of bone*** |  |
| Chondromesenchymal hamartoma of chest wall: not reported in NETSARC |  |
| Adamantinoma | 9261/3 |
| Undifferentiated sarcomas of bone | 8830/3 |
| Undifferentiated pleomorphic sarcoma of bone | 8830/3 |
| Undifferentiated sarcoma | 8803/3 |
| Undifferentiated spindle cell sarcoma | 8801/3 |
| Undifferentiated epithelioid sarcoma | 8804/3 |
| Leiomyosarcoma of bone | 8890/3 |
| Synovial sarcoma of bone | 9040/3 |
| Rhabdomyosarcoma of the bone | See RMS above |
| BCOR Sarcoma of bone | 8803/3 |
| Myoepithelioma of bone | 8982/3 |
| Liposarcoma of bone | 8850/3 |
|  |  |
| ***Haematopoietic neoplasms of bone*** |  |
| Solitary plasmocytoma of bone : not reported in NETSARC | 9731/3 |
| Primary non Hodgkin's lymphoma of bone : not reported in NETSARC | 9591/3 |
| Langerhans cell histiocytosis | 9752/1 |
| Erdheim Chester disease : not reported in NETSARC | 97550/1 |
| Rosai Dorfman disease: not reported in NETSARC | 8818/0 |

__________________________________________________________________________________
