# Supplementary material for: Nationwide incidence of sarcomas and connective tissue tumors of intermediate malignancy over four years using an expert pathology review network
Source: PLoS One. 2021 Feb 25;16(2):e0246958. doi: 10.1371/journal.pone.0246958 (PMC7906477; doi:10.1371/journal.pone.0246958)
Supplement: S3 Table — (DOCX) [file pone.0246958.s003.docx]

**S3 Table. Groups of histotypes presented in the tables.**

**_____________________________________________________________________________________________________________________**

**Groups Individual histotypes gathered in the group**

**Soft tissue & visceral sarcomas**

**Adipocytic tumours** All liposarcomas

ALT/WD LPS Atypical lipomatous tumour, well-differentiated liposarcoma

Myxoid round cell LPS Liposarcoma- myxoid, Liposarcoma round cell

**Fibroblastic and myofibroblastic tumours All histotypes**

Solitary fibrous tumour Solitary fibrous tumour, High risk solitary fibrous tumour

**So-called fibrohistiocytic tumours All histotypes**

**Vascular tumours All histotypes**

**Smooth muscle (SM) tumours All histotypes**

Leiomyosarcoma Leiomyosarcoma NOS, Leiomyosarcoma-differentiated, Leiomyosarcoma- poorly differentiated

**Skeletal muscle sarcoma (RMS) All histotypes**

Embryonal Rhabdomyosarcoma (ERMS) ERMS- botryoid type, ERMS usual type, ERMS spindle cell (8910/3)

**Peripheral nerve sheath tumor All histotypes**

Malignant Peripheral nerve sheath tumor

(MPNST) MPNST-epithelioid type, MPNST-usual type, Malignant peripheral nerve sheath tumour

**Tumors of uncertain differentiation not considered is as a single group**

Myoepithelioma, Myoepithelial carcinoma,

mixed tumor Myoepithelioma, Myoepithelial carcinoma, mixed tumor

Synovial sarcoma (SyS) SyS - NOS, SyS-biphasic, SyS-monophasic, SyS-poorly differentiated

Epithelioid sarcoma Epithelioid sarcoma, Undifferentiated epithelioid sarcoma

PECOMA including angiomyolipoma PECOMA-NOS, Malignant PECOMA

Undifferentiated sarcomas Undifferentiated pleomorphic sarcoma, Undiff sarcomas & US NOS, Undifferentiated spindle cell sarcomas.

**Bone sarcomas**

**Chondrosarcomas**  Central atypical cartilaginous tumour, chondrosarcoma grade 1, central chondrosarcoma grade 2 and 3,

Chondrosarcoma NOS, Peripheral chondrosarcoma, Periosteal chondrosarcoma, Clear cell chondrosarcoma, Mesenchymal chondrosarcoma, dedifferentiated chondrosarcoma

**Osteosarcoma** Low grade central osteosarcoma, low-grade central osteosarcoma, Dedifferentiated low grade central

osteosarcoma, Osteosarcoma, Osteosarcoma NOS, Conventional osteosarcoma, Osteoblastoma-like osteosarcoma, Telangiectatic Osteosarcoma, Small cell osteosarcoma, parosteal osteosarcoma, dedifferentiated parosteal osteosarcoma, periosteal osteosarcoma, high-grade surface osteosarcoma.

**Parosteal osteosarcoma** Parosteal osteosarcoma, dedifferentiated parosteal osteosarcoma

All **undifferentiated sarcoma of bone**  Undifferentiated pleomorphic sarcoma, Undifferentiated sarcoma, Undifferentiated spindle cell sarcoma Undifferentiated epithelioid sarcoma

**Uterine sarcoma**

Endometrial stromal sarcoma, low grade Endometrial stromal nodule, Endometrial stromal sarcoma, Endometrial stromal sarcoma-low grade

Leiomyosarcoma of the uterus

_______________________________________________________________________________________________________________________________
