# Supplementary figures and images for: Nationwide incidence of sarcomas and connective tissue tumors of intermediate malignancy over four years using an expert pathology review network
Source: PLoS One. 2021 Feb 25;16(2):e0246958. doi: 10.1371/journal.pone.0246958 (PMC7906477; doi:10.1371/journal.pone.0246958)

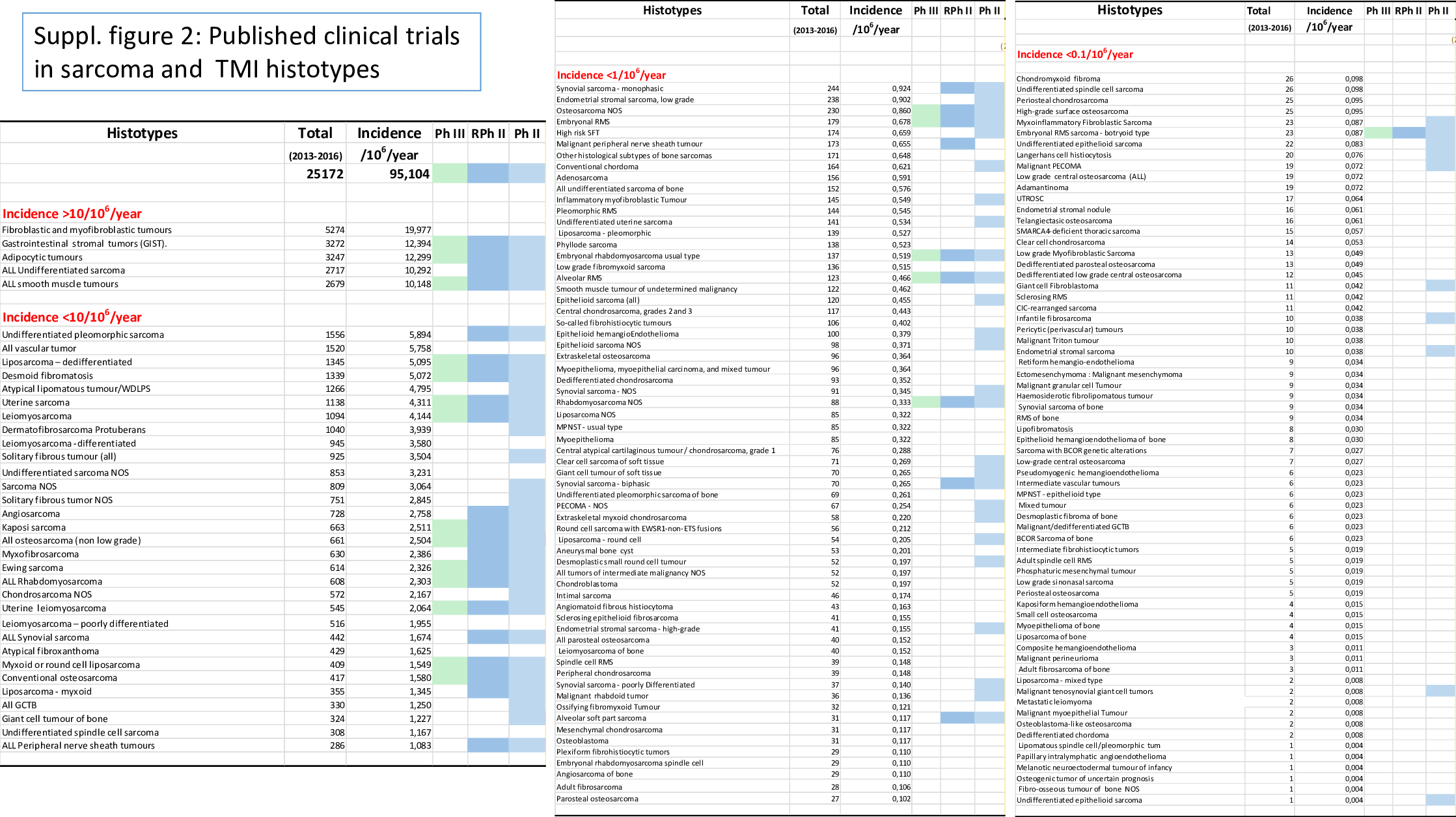

Supplement: S1 Fig — Tabular presentation of different sarcoma histotypes and groups of histotypes by decreasing order together with the documented published clinical trials in Pubmed. If phase III clinical trials are published, the box is highlighted in light green, if randomized phase II trials are published the box is highlighted in dark blue, if uncontrolled phase II trials are published the box is highlighted in light blue. Histotypes were considered individually (e.g. monophasic synovial sarcoma) or globally (e.g. all synovial sarcoma). (TIF) [file pone.0246958.s004.tif]

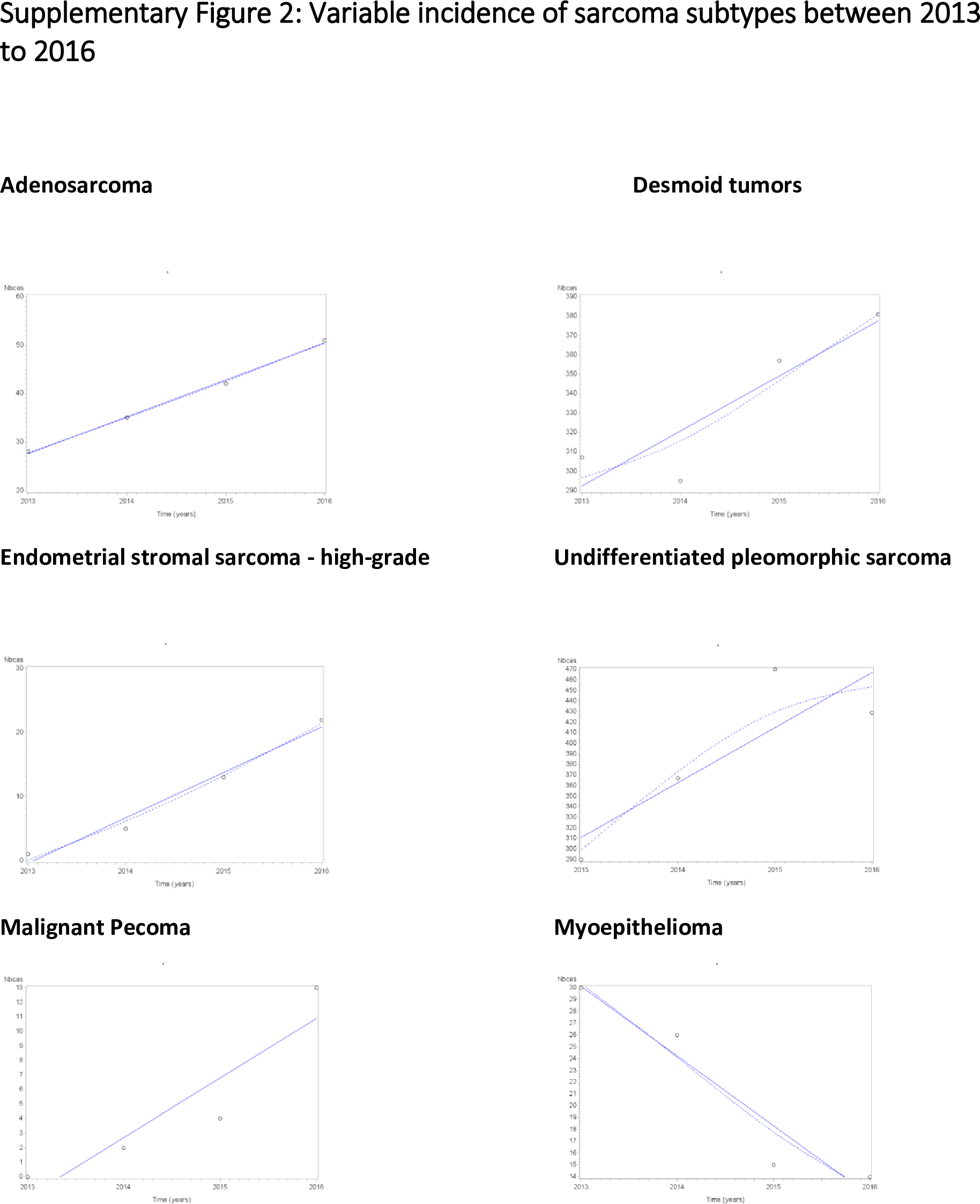

Supplement: S2 Fig — Presentation of the yearly variation of six different histotypes with significantly variable incidence in the period of observation. (TIF) [file pone.0246958.s005.tif]
