# Supplementary material for: Nationwide incidence of sarcomas and connective tissue tumors of intermediate malignancy over four years using an expert pathology review network
Source: PLoS One. 2021 Feb 25;16(2):e0246958. doi: 10.1371/journal.pone.0246958 (PMC7906477; doi:10.1371/journal.pone.0246958)
Supplement: Attachment — Submitted filename: Round 2.docx [file pone.0246958.s007.docx]

Major point

Authors wrote: “We investigated then the variability of the yearly incidence of these different tumors in the database. The analysis of variance of the observed incidence indicated a significant interaction between time and histology”. However, the analysis of variance is not described in Statistical Analyses

Minor points

| Where | It is | I would have written instead |
| --- | --- | --- |
| Abstract | The 2 later incidence groups | The 2 latter incidence groups |
| Introduction | INCa | INCA |
|  | … may not treated according to | … may not be treated according to |
| NETSARC+ network | French National Cancer institute | French National Cancer Institute |
| RREPS/NETSARC Database | MDTB | Acronym never defined |
| Results | This later group a group is not | This latter group is not |
