# Supplementary material for: Nationwide incidence of sarcomas and connective tissue tumors of intermediate malignancy over four years using an expert pathology review network
Source: PLoS One. 2021 Feb 25;16(2):e0246958. doi: 10.1371/journal.pone.0246958 (PMC7906477; doi:10.1371/journal.pone.0246958)
Supplement: Attachment — Submitted filename: Response to reviewers R3.docx [file pone.0246958.s009.docx]

**Response to reviewers**

PONE-D-20-18848R1
Nationwide incidence of sarcomas and connective tissue tumors of intermediate malignancy over four years using an expert pathology review network.
PLOS ONE

Dear Dr. Blay,

Thank you for submitting your manuscript to PLOS ONE. After careful consideration, we feel that it has merit but does not fully meet PLOS ONE’s publication criteria as it currently stands. Therefore, we invite you to submit a revised version of the manuscript that addresses the points raised during the review process.

The manuscript has been clearly improved. However, please carefully address further comments from reviewer 1 and minor additional comments  from reviewer 2.

Please submit your revised manuscript by Dec 12 2020 11:59PM. If you will need more time than this to complete your revisions, please reply to this message or contact the journal office at [plosone@plos.org](mailto:plosone@plos.org). When you're ready to submit your revision, log on to <https://www.editorialmanager.com/pone/> and select the 'Submissions Needing Revision' folder to locate your manuscript file.

Please include the following items when submitting your revised manuscript:

- A rebuttal letter that responds to each point raised by the academic editor and reviewer(s). You should upload this letter as a separate file labeled 'Response to Reviewers'.
- A marked-up copy of your manuscript that highlights changes made to the original version. You should upload this as a separate file labeled 'Revised Manuscript with Track Changes'.
- An unmarked version of your revised paper without tracked changes. You should upload this as a separate file labeled 'Manuscript'.

If you would like to make changes to your financial disclosure, please include your updated statement in your cover letter. Guidelines for resubmitting your figure files are available below the reviewer comments at the end of this letter.

If applicable, we recommend that you deposit your laboratory protocols in protocols.io to enhance the reproducibility of your results. Protocols.io assigns your protocol its own identifier (DOI) so that it can be cited independently in the future. For instructions see: [http://journals.plos.org/plosone/s/submission-guidelines#loc-laboratory-protocols](https://smex-ctp.trendmicro.com:443/wis/clicktime/v1/query?url=http%3a%2f%2fjournals.plos.org%2fplosone%2fs%2fsubmission%2dguidelines%23loc%2dlaboratory%2dprotocols&umid=aedf7725-5a4e-4d76-b61e-9c5cf027b39b&auth=88b027512158546030ba1422e012c48ffde03353-8378c91c97443179874a95d421e40af21f6a30dc)

We look forward to receiving your revised manuscript.

Kind regards,

Sandro Pasquali, M.D., Ph.D.
Academic Editor
PLOS ONE


Reviewers' comments:

Reviewer's Responses to Questions

**Comments to the Author**

1. If the authors have adequately addressed your comments raised in a previous round of review and you feel that this manuscript is now acceptable for publication, you may indicate that here to bypass the “Comments to the Author” section, enter your conflict of interest statement in the “Confidential to Editor” section, and submit your "Accept" recommendation.

Reviewer #1: (No Response)

Reviewer #2: All comments have been addressed

Reviewer #4: All comments have been addressed

2. Is the manuscript technically sound, and do the data support the conclusions?

The manuscript must describe a technically sound piece of scientific research with data that supports the conclusions. Experiments must have been conducted rigorously, with appropriate controls, replication, and sample sizes. The conclusions must be drawn appropriately based on the data presented.

Reviewer #1: Yes

Reviewer #2: Partly

Reviewer #4: Yes

3. Has the statistical analysis been performed appropriately and rigorously?

Reviewer #1: Yes

Reviewer #2: Yes

Reviewer #4: Yes

4. Have the authors made all data underlying the findings in their manuscript fully available?

The [PLOS Data policy](http://www.plosone.org/static/policies.action#sharing) requires authors to make all data underlying the findings described in their manuscript fully available without restriction, with rare exception (please refer to the Data Availability Statement in the manuscript PDF file). The data should be provided as part of the manuscript or its supporting information, or deposited to a public repository. For example, in addition to summary statistics, the data points behind means, medians and variance measures should be available. If there are restrictions on publicly sharing data—e.g. participant privacy or use of data from a third party—those must be specified.

Reviewer #1: No

Reviewer #2: Yes

Reviewer #4: Yes

5. Is the manuscript presented in an intelligible fashion and written in standard English?

PLOS ONE does not copyedit accepted manuscripts, so the language in submitted articles must be clear, correct, and unambiguous. Any typographical or grammatical errors should be corrected at revision, so please note any specific errors here.

Reviewer #1: Yes

Reviewer #2: No

Reviewer #4: Yes

**6. Review Comments to the Author

Please use the space provided to explain your answers to the questions above. You may also include additional comments for the author, including concerns about dual publication, research ethics, or publication ethics. (Please upload your review as an attachment if it exceeds 20,000 characters)**

**Reviewer #1: Thank you for the revised version of the paper which has greatly improved. However, some issues remain incomplete or to clarify.
Abstract** : **Conclusion: ... and that tumors with an incidence<106 /year have a much lower access to clinical trials. I think you mean with an incidence <1/106**

Answer The reviewer is absolutely right. We have made the appropriate modifications in the revised version

***The RREPS/NETSARC Database
The authors should better describe the quality of the DB including the completeness of the follow-up information which are essential to define the prevalence. Could the authors expand how they ensure the completeness of the follow-up? how life status information are collected? is there any indicator of lost to follow-up or any other?***

Answer : This is indeed an important point. The database is not systematically completed for follow-up by the CRA, as it would be in a clinical trial, but all baseline and first therapeutic informations are completed until the end of the first line treatment. This includes alll pathology reviews, which are therefore as presented , the final diagnoses. 65% of the patients have at least 6 months follow-up, and the median follow-up of the series was 17 months in the recent publications of the same dataset (25). Importantly, since 2019, the information (survival and all treatments) from the nationwide database of the social security system (SNDS, <https://www.snds.gouv.fr/SNDS/Accueil> ) is used to update the latest survival information of these patients as part of the Health datahub Deepsarc project (https://www.health-data-hub.fr/outil-de-visualisation), now ensuring a more exhaustive follow-up information. These points were added in the revised R2 version of the Ms.

**Presentation of the data
I do agree that the current table should not include the WHO codes. However, this is a key part of the material and methods selection since an article should include all the information to replicate the study. Please add the codes as supplementary Table.**

**Answer :** We added the WHO codes (2013, and whe relevant 2020) as a supplementary Table 1 in the revised R2 version.

**“The number of patients for each individual histological subtype of sarcoma or TIM per year, from 2013 to 2016, is therefore presented in these tables. To facilitate the comparison with other databases using previous classifications, the incidence for groups of tumors are also presented in the tables, when they are clinically relevant (e.g. uterine sarcoma). We also considered that it was still useful to present each individual histotype, (e.g. WDLPS) and groups of histotypes when clinically relevant (e.g. WD and DDLPS, or even liposarcoma, leiomyosarcomas). Conversely, when a grouping was not clinically meaningful in clinical routine (e.g. “fibroblastic and myofibroblastic tumors” in table 1) we did not consider this a distinct entity.”**

**This is still unclear. The authors should clarify what they count as a single entity. Please add a column on the left hand site of Table 1 and Table 2 indicating what was counted as single entity (N=150). It is not clear also why some grouping have the incidence and others not. it does not seem to depend on the clinical relevance because fibroblastic and myofibroblastic tumours have incidence data in Table 1. Kindly clarify.**

**Answer :** We agree that it is important to clarify the grouping. We have therefore added a supplementary Table 2 indicating all the groups which were used in the present report to facilitate cthe comparision with subsequent data sets.

Incidence
It is a pity not be able to separate TIM from malignant sarcomas. The authors used the WHO classifications which provides information about the sarcoma behaviour. Thus, the authors should clarify why it is not possible to distinguish TIM and malignant sarcomas. This goes back to the issues of understanding the codes used for the analyses.

**Answer** : To distinguish better TIM from sarcomas, we have added a colour code ( blue for TIM in table 4) in the revised version. We hope that this will facilitate the lecture.

Incidence of individual histotypes and published clinical trials.
“..... 14 of 35 (40%) histotypes with an incidence >1/10 6 had a dedicated phase III study vs 6 of 130 (4.6%) histotypes for sarcomas with a incidence <1/10 6 (p<10 -6 ). 20100 (79,7%) patients of the database had a specific histotype for which no phase III trial had been reported. 21 of 35 (60%) histotypes with an incidence >1/10 6 had a dedicated randomized phase II study vs 10 of 129 (7.7%) histotypes for sarcomas with a incidence <1/10 6 (p<10 -10 ))”

The authors should clarify whether the histotypes for sarcomas with a incidence <1/106 are 129 or 130.

Answer : We thank the reviewer for the careful. This mistale was corrected , the right number is N=130. This has been modified accordingly in the revised version R2.

Table 1. please check the incidence for Embryonal RMS and Myoepithelioma, myoepithelial carcinoma, & mixed tumour. The incidence of the histotype sum up differently from the incidence reported.

Answer : the sums were wrong and were corrected in the R2 version. We thank the reviewer for a careful attention

**Reviewer #2: Review Comments to the Author

Please use the space provided to explain your answers to the questions above. You may also include additional comments for the author, including concerns about dual publication, research ethics, or publication ethics. (Please upload your review as an attachment if it exceeds 20,000 characters) (Limit 100 to 20000 Characters)

Please see my comments in the attached file**

**Reviewer #4: The revised version addressed the points risen.
This work will represent the benchmark for future epidemiological studies.**

Answer : We thank the reviewer for these kind words

7. PLOS authors have the option to publish the peer review history of their article ([what does this mean?](https://smex-ctp.trendmicro.com:443/wis/clicktime/v1/query?url=https%3a%2f%2fjournals.plos.org%2fplosone%2fs%2feditorial%2dand%2dpeer%2dreview%2dprocess%23loc%2dpeer%2dreview%2dhistory&umid=aedf7725-5a4e-4d76-b61e-9c5cf027b39b&auth=88b027512158546030ba1422e012c48ffde03353-5bafbf68aaf640fe76af99b7cbef09e23f96e8ff)). If published, this will include your full peer review and any attached files.


If you choose “no”, your identity will remain anonymous but your review may still be made public.


**Do you want your identity to be public for this peer review?** For information about this choice, including consent withdrawal, please see our [Privacy Policy](https://smex-ctp.trendmicro.com:443/wis/clicktime/v1/query?url=https%3a%2f%2fwww.plos.org%2fprivacy%2dpolicy&umid=aedf7725-5a4e-4d76-b61e-9c5cf027b39b&auth=88b027512158546030ba1422e012c48ffde03353-7f9ee7908c4ea955affaa7ce1d056bf07f42fbf0).

Reviewer #1: No

Reviewer #2: **Yes:** Giuseppe Mastrangelo

Reviewer #4: **Yes:** Salvatore Lorenzo Renne
